# Multicenter Usability Evaluation and Co-Development of a Digital Decision-Support Tool for Labor Triage: Mixed Methods Study

**DOI:** 10.2196/87589

**Published:** 2026-07-23

**Authors:** Mariana Tome, Xavier Laurent, Kristiyan Georgiev, John Tolladay, Sarah Collins, Deborah Hedgecott, Lyuba V Bozhilova, Jane E Hirst, Lawrence Impey, Antoniya Georgieva

**Affiliations:** 1 Nuffield Department of Women's & Reproductive Health, Oxford Labour Monitoring, University of Oxford, Nuffield Department of Women's & Reproductive Health, University of Oxford, Oxford, England, OX3 9DU, United Kingdom, 1 01865 221004; 2 Artificial Intelligence (AI) Competency Centre, University of Oxford, Oxford, England, United Kingdom; 3 The George Institute for Global Health, School of Public Health, Imperial College London, London, England, United Kingdom

**Keywords:** cardiotocography, fetal monitoring, usability testing, clinical decision support systems, labor management, health care technology, co-development methodology, maternity care, digital health, labor triage, user-centered design, implementation, data-based tools in health care

## Abstract

**Background:**

Digital decision-support tools for labor care remain limited, with few technologies successfully addressing the complex, time-sensitive decisions required during labor triage. Fit4Labour is a clinician-facing, data-driven research tool, currently under development, that combines computerized cardiotocography interpretation with maternal and fetal risk factors to generate individualized risk scores at labor onset. Its primary aim is to support clinicians in identifying fetuses who may require closer monitoring or expedited delivery, while simultaneously providing reassurance in low-risk cases. By promoting consistent communication and timely escalation of care, the Fit4Labour tool seeks to strengthen clinical decision-making. Understanding and addressing usability and implementation barriers will be critical to its adoption in clinical practice.

**Objective:**

This study aims to evaluate whether a digitally co-developed labor decision-support tool (Fit4Labour) maintains usability and implementation readiness across NHS hospitals with differing clinical contexts.

**Methods:**

We conducted a convergent parallel mixed methods study in 3 United Kingdom hospitals (December 2022 to May 2025). Phase 1 involved iterative co-development with midwives and doctors at Oxford University Hospitals NHS Foundation Trust; Phase 2 validated the locked version at Birmingham Women’s and Children’s NHS Foundation Trust and Buckinghamshire Healthcare NHS Trust. Participants completed scenario-based usability sessions evaluated with the System Usability Scale (SUS) and Single Ease Question (SEQ), and task completion time, followed by focus groups and interviews analyzed thematically.

**Results:**

Twenty-six health care professionals participated: 12 in co-development (7 midwives, 5 doctors) and 14 in validation (8 midwives, 6 doctors) phases. During co-development at Oxford, the tool met the “excellent” usability threshold (mean SUS 82.1, SD 12.3), indicating readiness for the validation phase. The locked version (v4.0) independently met the “excellent” threshold at both validation sites (combined mean SUS 85.8, SD 10.2; Birmingham 80.7, SD 10.8; Buckinghamshire 90.8, SD 7.2). Task completion times were comparable across validation sites (Birmingham 10.3, SD 1.6 min; Buckinghamshire 9.2, SD 1.9 min), while SEQ scores were consistently high across all scenarios (mean 6.1/7, SD 0.8). Thematic analysis identified 12 themes within 3 domains: clinical integration and workflow, technology adoption and implementation, and patient safety and decision-making. Participants described the Fit4Labour tool as a supportive tool, “like a co-pilot,” improving confidence in decisions with the potential to aid triage assessment. Perceived limitations included an incomplete risk factor profile and the need for minor technical adjustments or integration with existing hospital systems.

**Conclusions:**

Through systematic co-development, the Fit4Labour tool met the established usability benchmark at 2 independent NHS hospitals with markedly different clinical contexts. Clinicians viewed the tool as a supportive aid providing a shared language for risk communication and enhanced decision-making while preserving clinical autonomy. Whether these usability findings translate to improved clinical outcomes in real-world practice requires prospective evaluation.

## Introduction

### Overview

Health care systems worldwide are undergoing rapid digital transformation, with clinical decision-support tools increasingly recognized as valuable tools for improving patient safety and care quality [1-5]. This transformation is particularly important in maternity care, where time-sensitive decisions during labor have profound consequences for both maternal and neonatal outcomes [6-8]. Yet, successful implementation depends not only on algorithmic accuracy, but also on user-centered design: tools must present information clearly, fit seamlessly into workflows, and support rather than disrupt decision-making [9-13].

### Implementation Challenge in Health Care Technology

Traditional development models in health care technologies often involve limited user engagement until late validation stages, resulting in tools that perform well in controlled settings (“work as imagined”) but fail in the reality of busy clinical environments (“work as done”) [14-16]. Insufficient investment of time and resources in co-design and usability testing remains a major barrier to adoption. Systematic reviews consistently demonstrate that poor usability is linked to low uptake, which in turn can pose patient safety risks [17-19].

Maternity care poses additional unique challenges as clinicians often manage multiple patients simultaneously with diverse risk profiles, while maintaining high safety standards [20,21]. Front-line clinical staff (eg, midwives and junior doctors) in the United Kingdom (UK) National Health System (NHS) are often key decision-makers in triage, and hold unique insights into workflow barriers; however, their perspectives are frequently underrepresented during the design of new technologies [22,23].

### Challenges in Labor Care

Fetal monitoring can be performed using intermittent auscultation or cardiotocography. Cardiotocography, a graphical record of fetal heart rate and uterine contractions over time, remains the standard tool for monitoring during labor [8,24]. However, cardiotocography interpretation demands specialist expertise, and yet, the same trace can be classified differently, which may lead to suboptimal decisions [25-27].

Labor triage involves the rapid assessment of maternal and fetal well-being at the onset of labor to determine appropriate monitoring, the appropriate place for birth, and whether urgent intervention (such as expedited delivery) may be required [22,24,28]. These early decisions shape the entire labor care pathway. Published investigations repeatedly identified that failures during intrapartum monitoring are associated with delays in both decision-making and escalation of care [29,30]. At the same time, modern maternity care increasingly emphasizes shared decision-making, requiring clinicians to communicate individualized risk information effectively to support women’s autonomy [7,24,31].

### The Fit4Labour Tool Innovation

**Figure 1.**
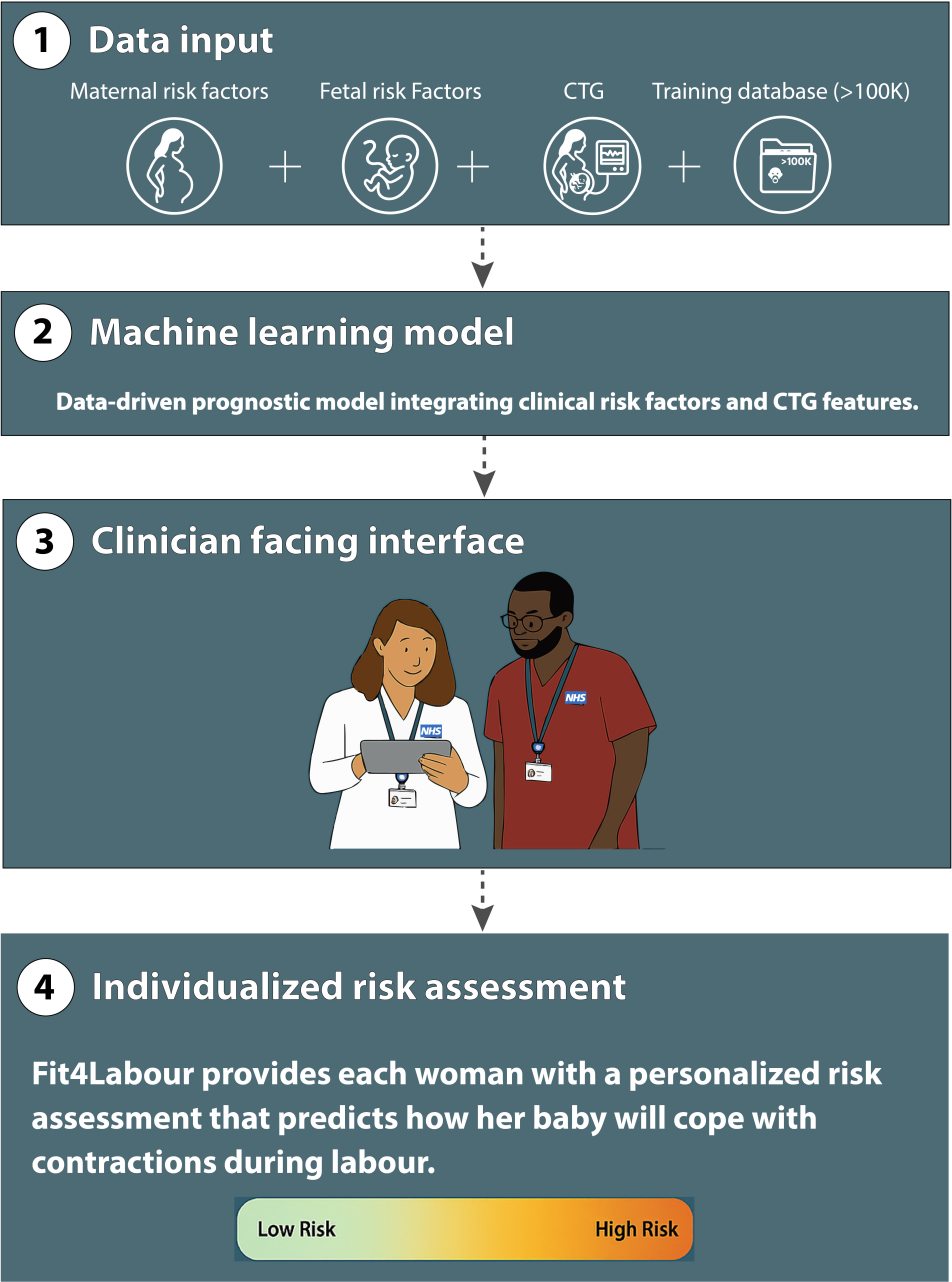
The Fit4Labour tool clinical workflow pathway from initial risk assessment to generation of the risk report. The diagram illustrates how the tool integrates into existing maternity workflows, combining maternal and fetal risk factors with computerized analysis of the cardiotocography trace to support triage, escalation, and communication of risk. CTG: cardiotocography.

The Fit4Labour tool (University of Oxford) was designed as a clinician-facing digital system to address these challenges by combining maternal and fetal risk factors (eg, maternal age, diabetes, and meconium-stained liquor) with computerized cardiotocography analysis to generate individualized risk scores indicating the likelihood of fetal compromise during labor (Figure 1). Unlike earlier computerized cardiotocography systems that rely mainly on human pattern recognition, the Fit4Labour tool applies a prognostic modeling approach that integrates multiple clinical risk factors with cardiotocography data to produce standardized, evidence-based predictions of adverse outcomes at delivery [32,33]. Developed by the Oxford Labor Monitoring Group through multidisciplinary, user-centered co-design involving clinicians, engineers, and patient representatives, the tool draws on a dataset of over 147,000 cardiotocographs linked to birth outcomes and prioritizes intuitive use and clear risk communication to support clinicians’ intrapartum decision-making [34].

This multicenter convergent parallel mixed methods study aimed to evaluate whether the Fit4Labour tool, developed through intensive co-development at a single NHS hospital, maintained usability and implementation readiness across 2 NHS hospitals with differing clinical contexts.

## Methods

### Study Design and Conceptual Framework

We conducted a multicenter convergent parallel mixed methods study over a two-and-a-half-year period (December 2022-May 2025) to assess the usability and implementation readiness of the Fit4Labour decision-support tool. Quantitative usability metrics were combined with qualitative user experience data, analyzed in parallel and then systematically integrated. This approach allowed us to explore both the measurable performance and clinicians’ lived experience, providing a comprehensive understanding of usability and potential implementation barriers.

**Figure 2.**
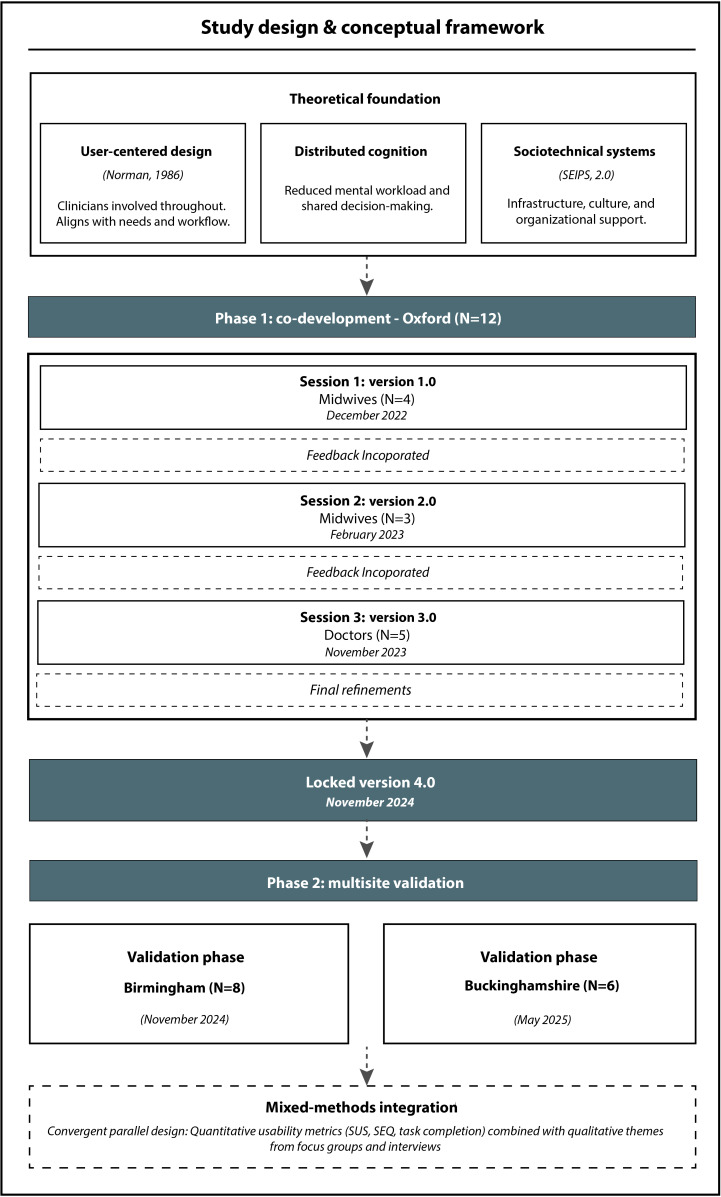
Conceptual framework and study design showing the Fit4Labour tool version iterations. SEIPS: Systems Engineering Initiative for Patient Safety. SEQ: Single Ease Question; SUS: System Usability Scale.

The study was guided by human factors engineering principles and we used 3 theoretical approaches (Figure 2). First, user-centered design entails involving midwives and doctors throughout development [9]. We designed the tool based on user feedback, with the aim of aligning its features to clinicians’ cognitive workflows and practical needs. Second, Distributed Cognition recognized that clinical decision-making is shared across people, tools such as checklists or cardiotocography traces, rather than occurring solely in individual minds [35]. The Fit4Labour tool was designed to provide both midwives and doctors with the same risk information, creating a shared risk score that supports team coordination and collective decision-making. Third, Sociotechnical Systems emphasized that technology operates within complex systems of people, processes, and environments [12,36]. This framework guided our assessment of whether participating hospitals had the necessary infrastructure, culture, and organizational support to implement the Fit4Labour tool effectively.

The study was conducted in 2 phases. Phase 1 was an iterative co-development process at Oxford University Hospitals NHS Foundation Trust (OUH) (referred to as Oxford throughout the paper), where the tool was refined through multiple cycles of usability testing and direct user feedback. Phase 2 involved multisite validation at Birmingham Women’s and Children’s NHS Foundation Trust (referred to as Birmingham throughout the paper) and Buckinghamshire Healthcare NHS Trust (referred to as Buckinghamshire), where the locked prototype version of the tool was evaluated to assess its usability and transferability across different organizational contexts.

### Study Sites

Hospitals were intentionally selected to capture diverse maternity services and populations within the UK National Health Service. OUH (Oxford) is a large university teaching hospital with a well-established research infrastructure and strong links to the University of Oxford, serving both urban and rural populations. BWC (Birmingham) is a specialist teaching hospital providing high-volume maternity services to an ethnically diverse population, while BHT (Buckinghamshire) is a medium-sized district general hospital broadly representative of typical NHS maternity care, reflecting the realities of routine clinical practice. Together, these sites enabled evaluation across varied demographics, clinical practices, and infrastructure levels, thereby enhancing the generalizability within the NHS hospitals and ethnically diverse populations.

### The Fit4Labour Tool

The clinician-facing Fit4Labour tool is a web-based application that runs on tablet devices or computers and can communicate with electronic maternity record systems. It integrates maternal and fetal risk factors with computerized cardiotocography analysis to produce color-coded risk categories (green=low, amber=moderate, red=high) accompanied by a numerical score indicating the risk of severe fetal compromise. Severe compromise is defined as a composite of intrapartum stillbirth, neonatal death (<28 days), neonatal encephalopathy (including hypoxic-ischemic encephalopathy), or neonatal seizures.

The tool displayed time-related changes in risk that updated in real time as the cardiotocography recording progressed, alongside detailed computerized analyses of individual cardiotocography parameters including baseline heart rate variability, accelerations, and decelerations. Reports were generated at 20- and 60-minute intervals and included integrated note-taking and record-storage features to support clinical documentation, handover continuity, and audit processes.

### Study Objectives

The primary objective was to evaluate usability and acceptability of the Fit4Labour tool across 3 NHS sites using quantitative metrics (System Usability Scale, Single Ease Question, task completion times) and qualitative assessment of clinician perceptions. Secondary objectives were to explore organizational factors influencing transferability, examine how midwives and doctors perceived and interacted with the tool, assess its perceived potential to enhance patient care, and identify organizational and technical barriers that may impact adoption.

### Participants and Recruitment

Participants comprised qualified midwives and doctors currently working in the selected maternity services, with direct patient care responsibilities in labor triage or delivery settings, and the need for and experience in cardiotocography interpretation as part of their clinical role. Department managers distributed information sheets to recruit participants. Three factors determined sample size: Nielsen’s guidance for interface evaluation (5‐8 participants per group typically uncover 80%‐85% of usability issues in interface evaluation studies, a pattern following the diminishing returns where each additional participant identifies fewer new problems), thematic saturation requirements for qualitative analysis, and practical constraints of multisite recruitment [37]. Using a relatively small number of samples, our convergent mixed methods design prioritized depth of insight over statistical power, consistent with exploratory implementation research [38,39]. All participants received a small honorarium (Amazon voucher) for their time.

### Procedures

Between December 2022 and January 2024, the co-development phase took place in Oxford with participants testing successive prototypes in structured usability sessions. Feedback was sought on the user interface, layout, workflow, data entry, and risk visualization. Development followed a user-centered, multidisciplinary approach with iterative refinement over 4 versions at Oxford. Each version incorporated feedback from midwives and doctors, addressing user interface design, navigation, and risk visualization. Detailed version histories are provided in Table S3 in Multimedia Appendix 1. All design modifications were systematically logged, including user feedback prompting the change, implementation details, and impact assessment on overall functionality. Once usability goals, defined by high ease-of-use ratings, absence of major usability issues, and thematic saturation in qualitative feedback were achieved, the version was locked for validation testing. The locked version represented a stable version with no further interface or algorithmic modifications permitted during the validation phase. From November 2024 to May 2025, the locked version underwent prospective validation at Birmingham and Buckinghamshire. Participants followed the same protocol used at Oxford.

### Usability Testing Sessions

Sessions lasted between 60 and 120 minutes and were co-facilitated by a clinical researcher (MT) and an experimental psychologist (XL) with extensive experience in usability testing. All sessions were conducted in dedicated rooms to minimize interruptions and ensure a consistent testing environment.

Testing was done in 2 separate stages (Figure S1 in Multimedia Appendix 1). In the task-based evaluation, participants first viewed a standardized 5-minute introduction video explaining the tool’s purpose. The video did not include instructions on how to use the tool, as we sought to evaluate the user’s intuitive navigation (Multimedia Appendix 1 video). The users were then given the tool and were asked to enter data for 4 progressive clinical scenarios, simulating common clinic triage tasks. This included baseline clinical risk factor entry, report generation, note-taking, risk factor updates, and advanced report features (Table S1 in Multimedia Appendix 1). Each scenario lasted up to 5 minutes, with facilitators observing silently and recording task completion times, errors, and usability barriers. If a task could not be completed within the allocated time, participants were instructed to move on to the next task.

After completing each scenario, participants were asked to rate the task’s difficulty using the Single Ease Question (SEQ), a 7-point Likert scale (1=very difficult, 7=very easy). At the end of all tasks, they subsequently completed the System Usability Scale (SUS), a 10-item questionnaire generating an overall usability score from 0 to 100 (Figure S2 and S3 in Multimedia Appendix 1).

The second stage was the qualitative exploration, which involved profession-specific focus groups or, where necessary, individual interviews. Discussions followed a structured topic guide exploring ease of use, clinical relevance, workflow integration, training needs, communication implications, and impact on clinical autonomy. All sessions were audio-recorded and transcribed verbatim.

### Outcomes

Quantitative outcomes included SUS scores: values below 68 indicating below-average usability, between 68 and 80 indicating good usability, and above 80 indicating excellent usability [40,41]. SEQ ratings were used to assess ease of use following each clinical scenario. Lastly, task performance parameters were evaluated through completion times, error rates, and variability across participants. Qualitative outcomes captured themes relating to clinical integration, technology adoption, patient safety, and decision-making, drawing on data from focus groups and interviews.

### Analysis

#### Quantitative Analysis

Descriptive statistics were reported as mean (SD) or median (IQR) according to the data distribution that was assessed by the Shapiro-Wilk test. No inferential statistical testing was performed. This was a deliberate choice as the primary aim of this study was to establish readiness and understand patterns of usability performance within each phase, rather than test population-level hypotheses. Moreover, phase 1 and phase involved different participants, tool versions, and clinical sites, precluding between-phase comparison being attributed to a single factor. The sample size was deemed sufficient for thematic saturation in the qualitative data. Data analysis and graphical representation were performed using the R software (version 4.5.3; R Core Team).

#### Qualitative Analysis

Transcripts were analyzed inductively using Braun and Clarke’s 6-phase framework [42,43]. Three researchers (MT, XL, and DH) independently coded the transcripts, with consensus meetings to refine the coding framework and resolve any discrepancies. Themes were iteratively developed and mapped to usability and implementation domains. Saturation was defined as the absence of new themes across 2 consecutive coding rounds. NVivo software (Lumivero) was used for coding and data management.

#### Mixed Methods Integration

Quantitative and qualitative findings were integrated using a convergent parallel design [38]. Independent analyses were brought together in joint displays, with patterns categorized as convergence (alignment of findings), complementarity (mutual enrichment), or dissonance (contradictory insights). This approach enables a richer understanding of usability and implementation than either method alone.

### Ethical Considerations

This usability study was conducted independently of, but in parallel with, the Fit4Labour technology evaluation and proof-of-concept study (ethics reference: 22/SC/0097). As this study involved health care professionals evaluating a prototype tool in a simulated setting, and did not involve patients, patient data, or clinical decision-making, formal research ethics committee review was determined not to be required. This was confirmed and agreed with the principal investigator at each of the 3 participating sites prior to recruitment.The study was registered with, and formally endorsed by, the research governance team at each of the three participating sites prior to recruitment, and this determination was further confirmed and agreed with the principal investigator at each site. All participants provided informed consent, recorded at the start of each audio-recorded session, after receiving detailed information regarding the study objectives and procedures, including their right to withdraw at any time without giving a reason. No identifiable patient data were collected or used in this study, and audio recordings and transcripts were stored securely with access restricted to the research team. Participants received a small honorarium (an Amazon voucher) in recognition of their time. Funding for the usability testing was provided by a National Institute for Health and Care Research (NIHR) Invention for Innovation (i4i) Product Development Award (NIHR202117); the funder had no role in study design, data collection, analysis, or interpretation.

## Results

### Participant Characteristics

Twenty-six health care professionals were recruited across 3 NHS sites: 15 midwives (58%) and 11 doctors (42%). Twelve participants (7 midwives and 5 doctors) contributed to Phase 1 co-development at Oxford, and 14 participants (8 midwives and 6 doctors) to Phase 2 validation at Birmingham and Buckinghamshire. Most participants (22/26, 85%) were women. Participants represented a wide range of seniority, from junior staff to consultant midwives and obstetricians, broadly reflecting the professional mix of UK maternity care (Table S2 in Multimedia Appendix 1). Although 25 participants completed the scenario-based usability testing, 26 took part in the interviews.

### The Fit4Labour Tool Developments

The co-development phase at Oxford involved 3 iterative sessions, with each systematically incorporating users’ feedback and testing progressively refined versions of the Fit4Labour tool (Figure 2). The first session with midwives (n=4) evaluated version 1.0, followed by a second midwives’ session (n=3) a few months later testing version 2.0, and finally, version 3.0 was tested by doctors (n=5). This led to the final locked version 4.0, which was then tested in the 2 validation sites (Birmingham and Buckinghamshire). Detailed systematic documentation of design modifications is provided in Table S3 in Multimedia Appendix 1 and examples of user interface improvements in Figure S5 in Multimedia Appendix 1.

### Quantitative Findings

#### System Usability Scale (SUS)

**Figure 3.**
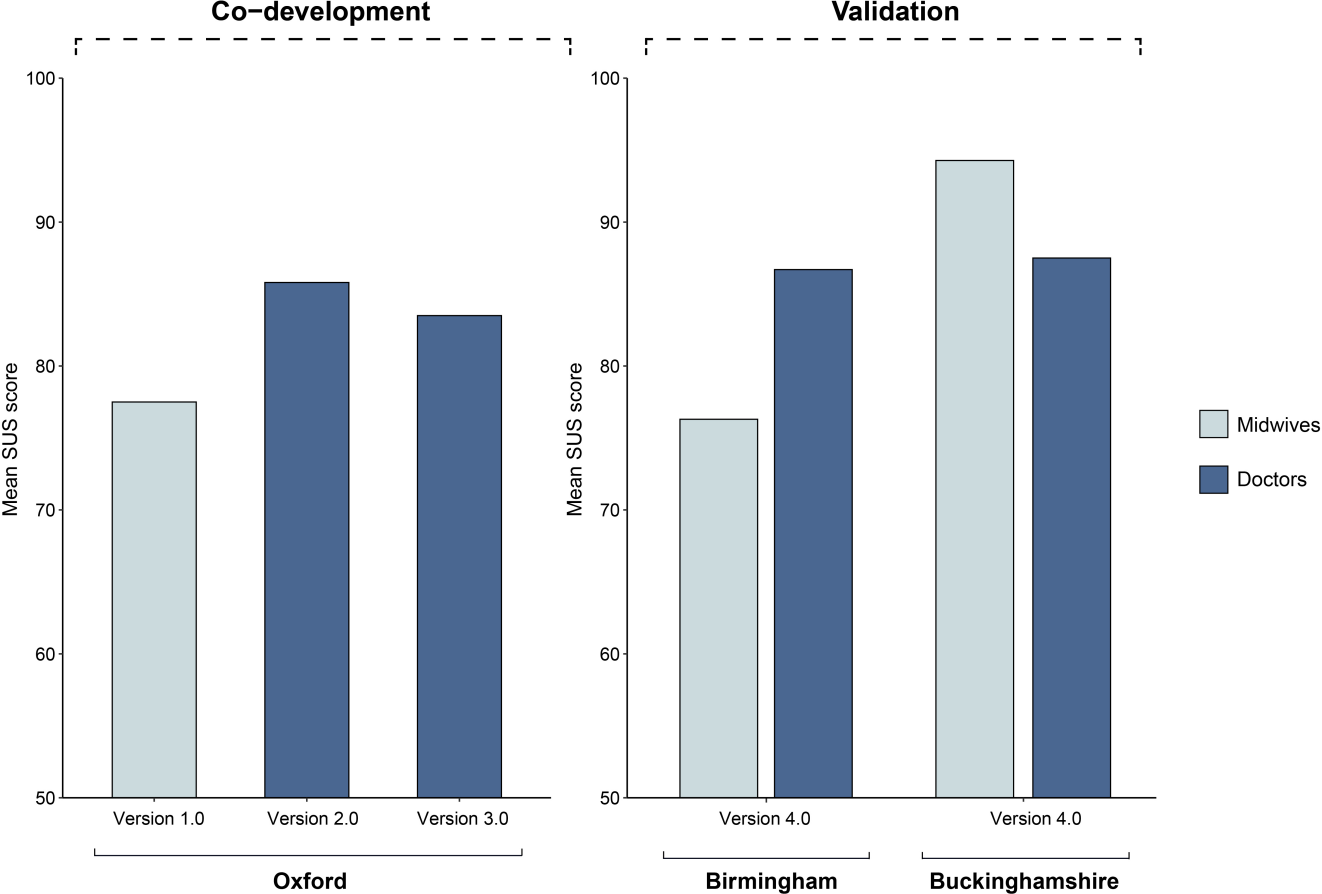
SUS scores by study phase and site. During co-development, scores reflect iterative refinement across successive versions. During validation, the locked version (v4.0) independently met the “excellent” threshold at both sites. SUS: System Usability Scale.

During the co-development at Oxford, the initial version (1.0) had a mean SUS of 77.5 (SD 15.1; rated as “good”), and by version 3.0 this had reached 83.5 (SD 9.1; rated as “excellent”). The co-development phase using the locked version (v4.0) had an overall mean SUS of 82.1 (SD 12.3) (Table 1, Figure 3), which indicated that the threshold for the locked version had been met (SUS>80).

**Table 1.** System Usability Scale scores by study phase and site. Learnability scores derived from System Usability Scale items 4 and 10.

Phase, site, and professional group	Participants, n	Version tested	SUS^b^ score, mean (SD)	Range	Learnability, mean (SD)	Ratings^c^
Co-development						
Oxford						
1st Midwives session	4	v1.0	77.5 (15.1)	62.5‐95	81.2 (16.1)	Good
2nd Midwives session	3	v2.0	85.8 (13.7)	70‐95	79.2 (19.1)	Excellent
Doctors	5	v3.0	83.5 (9.1)	72.5‐97.5	87.5 (12.5)	Excellent
Co-development total	12	v1.0‐3.0	82.1 (12.3)	62.5‐97.5	83.3 (14.4)	Excellent
Validation						
Birmingham						
Midwives	4	v4.0	76.3 (11.4)	65‐90	62.5 (22.8)	Good
Doctors	3		86.7 (6.6)	77.5‐92.5	75 (33.1)	Excellent
Site Subtotal	**7**		80.7 (10.8)	65‐92.5	67.9 (25.9)	Excellent
Buckinghamshire						
Midwives	3	v4.0	94.2 (5.1)	87.5‐100	95.8 (7.2)	Excellent
Doctors	3		87.5 (8.2)	77.5‐97.5	95.8 (7.2)	Excellent
Site subtotal	6		90.8 (7.2)	77.5‐100	95.8 (6.5)	Excellent
Validation total	13	v4.0	85.8 (10.2)	65‐100	80.8 (23.7)	Excellent
Overall study (all participants^d^)	25	v1.0‐4.0	83.8 (11.3)	62.5‐100	82 (19.5)	Excellent

b SUS: System Usability Scale.

c Interpretation thresholds: <68=below average, 68–80=good, >80=excellent.

d One Birmingham midwife observed scenarios only and participated solely in focus groups, resulting in n=26 total participants but n=25 for SUS assessments.

The final locked version 4.0 independently met the “excellent” usability threshold during validation testing at 2 distinct sites, with a combined mean SUS score of 85.8 (SD 10.2), but with some intersite variability (Birmingham 80.7, SD 10.8 vs Buckinghamshire 90.8, SD 7.2). The SD of 10.2 within this phase reflects a relatively narrow spread of individual scores, suggesting a more predictable and uniform user experience across participants and sites.

Learnability scores (SUS items 4 and 10) showed an overall mean of 82 (SD 19.5). Co-development mean scores were 83.3 (SD 14.4) (Oxford) and validation mean scores were 80.8 (SD 23.7). Buckinghamshire showed a mean of 95.8 (SD 6.5), while Birmingham showed greater variability at a mean of 67.9 (SD 25.9). Professional group differences were modest (doctors: mean 87.5, SD 12.5; midwives: mean 80.4, SD 15.9 at Oxford; Table 1).

#### Single Ease Question (SEQ)

**Figure 4.**
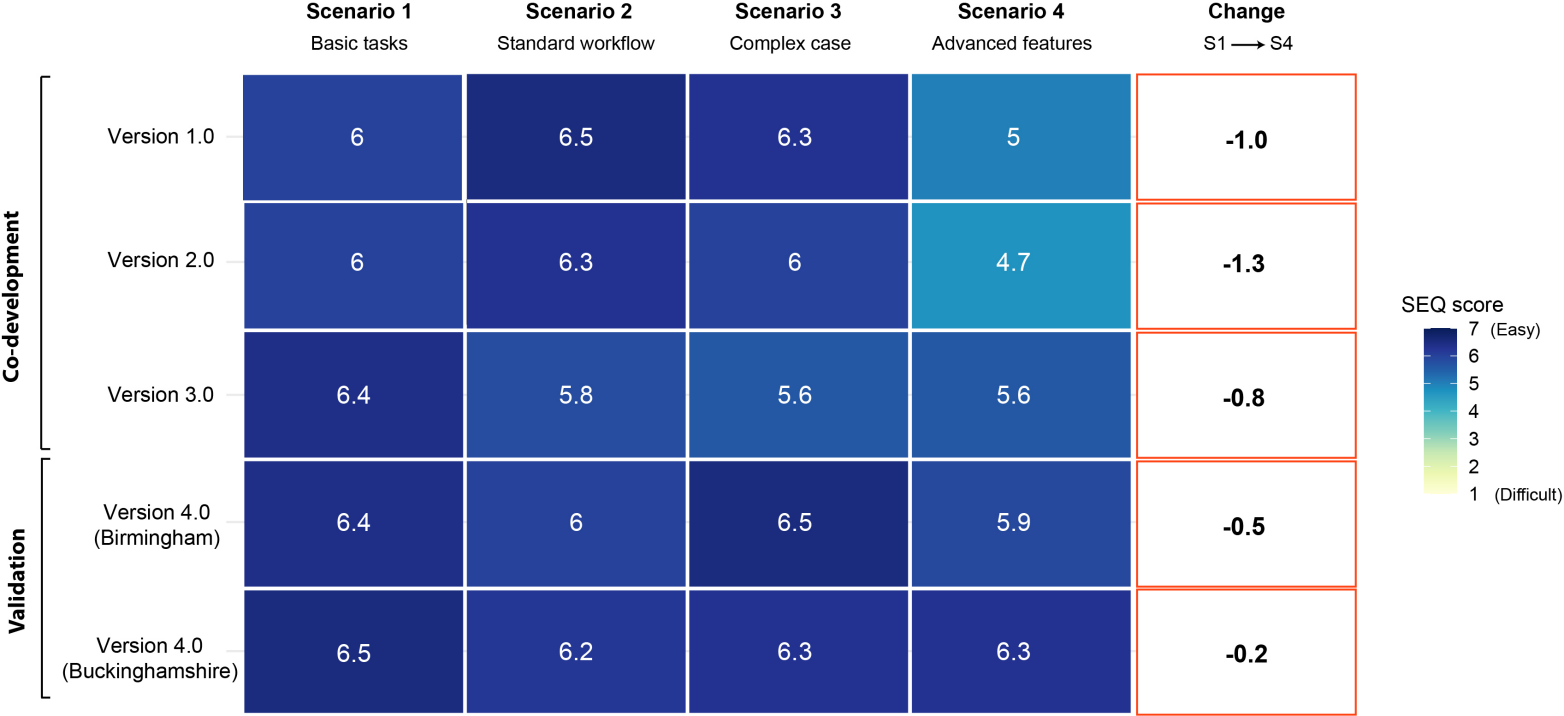
Heat map single ease question scores by study phase and scenario. Color-coded visualization of ease-of-use ratings (1=very difficult; 7=very easy) across scenarios*.* SEQ: Single Ease Question.

Task difficulty ratings demonstrated that participants perceived most scenarios as being “very easy” across all versions (mean SEQ 5.8‐6.3 for Scenarios 1‐3) (Table 2, Figure 4). Scenario 4, covering advanced functions, showed more variable ratings during co-development (v1.0 mean SEQ 5, SD 1.4; v2.0 mean SEQ 4.7, SD 1.2; and v3.0 mean SEQ 5.6, SD 0.7). In the validation phase, the locked version received SEQ ratings of mean 5.9 (SD 0.9) (Birmingham) and mean 6.3 (SD 0.8) (Buckinghamshire) for Scenario 4, indicating that advanced functions were perceived as easy to use. Overall, both validation sites demonstrated comparable ratings across all scenarios (Birmingham SEQ 6.2 vs Buckinghamshire SEQ 6.3), suggesting that version 4.0 had consistent perceived ease of use across scenarios.

**Table 2.** Single ease question scores by study phase and scenario. Single Easy Question Scale: 1=Very Difficult, 7=Very Easy.

Phase, site, and group	Participants, n	Fit4Labour version tested	Mean SEQ^a^ (SD)				
			Scenario 1	Scenario 2	Scenario 3	Scenario 4	Total
Co-development							
Oxford							
1st Midwives	4	v1.0	6 (0.8)	6.5 (0.6)	6.3 (0.5)	5.0 (1.4)	5.9
2nd Midwives	3	v2.0	6 (0)	6.3 (0.6)	6 (1)	4.7 (1.2)	5.8
Doctors	5	v3.0	6.4 (0.5)	5.8 (0.8)	5.6 (0.9)	5.6 (0.7)	5.9
Validation							
Birmingham							
Combined	7	v4.0	6.4 (0.7)	6.0 (0.9)	6.5 (0.5)	5.9 (0.9)	6.2
Buckinghamshire							
Combined	6	v4.0	6.5 (0.5)	6.2 (0.8)	6.3 (0.8)	6.3 (0.8)	6.3
Overall^b^	25	v4.0	6.3 (0.6)	6.1 (0.8)	6.1 (0.7)	5.7 (1)	6.1

a SEQ: Single Ease Question.

b One Birmingham midwife observed scenarios only and participated solely in focus groups, resulting in n=26 total participants but n=25 for SEQ assessments.

#### Task Efficiency and Errors

Cumulative task completion times in the co-development phase in Oxford ranged from 7 to 18 minutes, with a mean of 11.8 (SD 2.5) reflecting expected variation during refinements across successive prototype versions and iterative designs (Table S4 in Multimedia Appendix 1).

**Figure 5.**
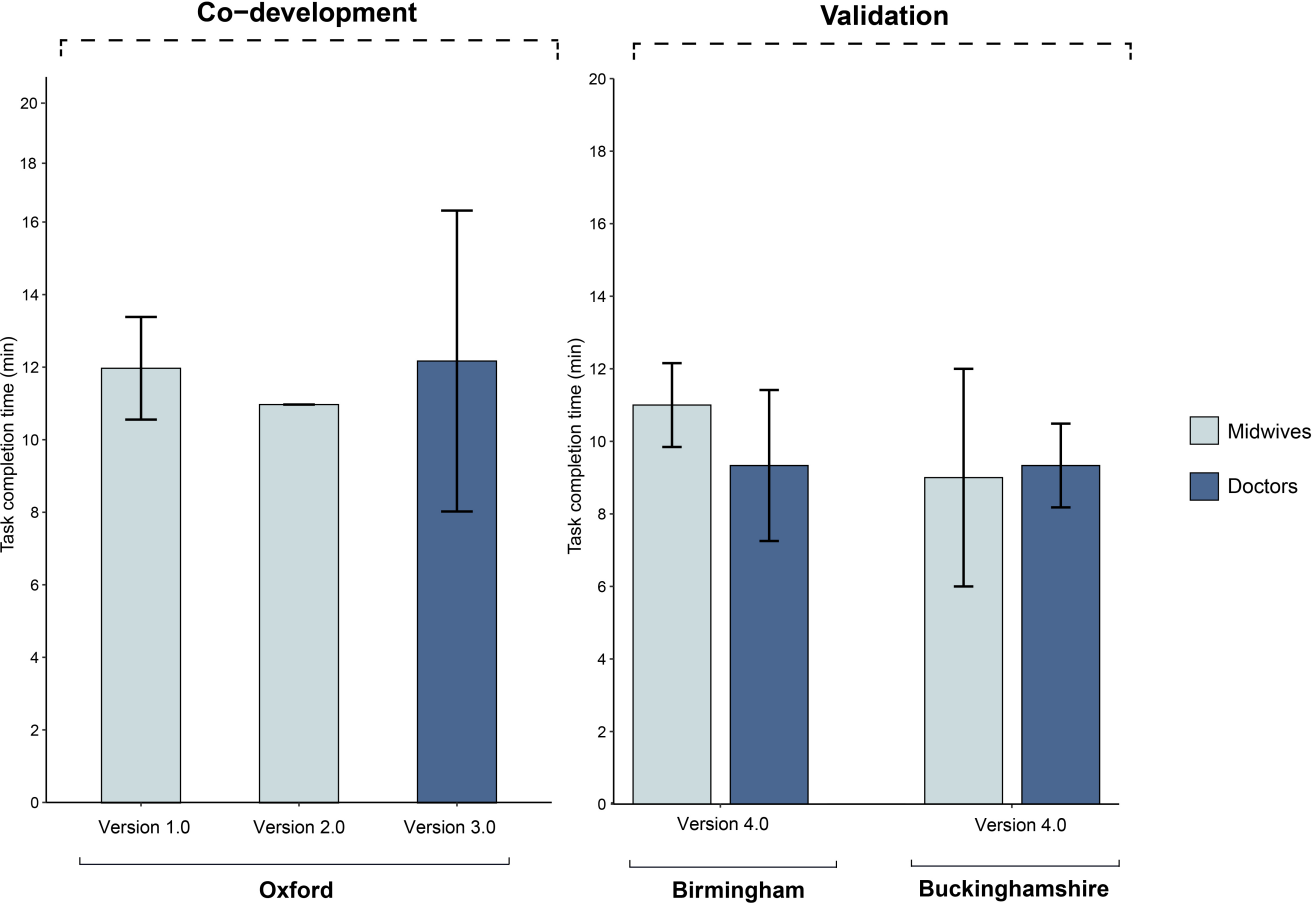
Task completion times (minutes) for all 4 simulated clinical scenarios completed sequentially during usability testing across study phases and sites. Each bar represents the total time taken to complete the entire set of 4 scenarios, not the time required to use the Fit4Labour tool in real clinical practice for 1 patient.

During the validation phase, the locked version 4.0 was completed in a mean of 9.8 (SD 1.8) minutes overall (Figure 5). Despite testing in different clinical environments and with different digital infrastructure, both validation sites had comparable completion times (Birmingham 10.3, SD 1.6 min and Buckinghamshire 9.2, SD 1.9 min), suggesting consistent and transferable usability of the locked version across sites.

#### Professional Group Comparisons

Usability was comparable between midwives and doctors. Doctors reported slightly higher SUS scores (mean 87.1, SD 8.2 vs mean 83, SD 13.8) and faster completion times (9.3 versus 10.1 min), but differences were modest. Midwives had higher variability in both. SEQ ratings were also very similar, indicating intuitive usability across professional groups.

### Qualitative Findings

Thematic analysis identified 12 themes grouped within 3 domains: clinical integration and workflow, technology adoption and implementation, and patient safety and decision-making (Table 3). All thematic summaries and supporting quotes are presented in the Supplementary Analysis 1‐3 and Figure S4 in Multimedia Appendix 1.

**Table 3.** Thematic framework showing the 3 domains and 12 themes identified through qualitative analysis across study sites. Summary of the 12 themes organized under 3 domains: clinical integration and workflow, technology adoption and implementation, and patient safety and decision-making.

Domain and theme number	Theme name	Description
Clinical integration and workflow		
1	Risk factors and clinical comprehensiveness	Need for more risk factor options (eg, IUGR^a^, reduced fetal movements) and complex combinations to improve accuracy and avoid underestimating risk.
2	Timeline and workflow integration	Report timing options (20 versus 60 min) and workflow challenges including room availability, resources, and staffing constraints.
3	Screening and triage effectiveness	Value in objective screening and triage with color-coded risk stratification (green, red, amber) for prioritizing care.
4	Escalation support for midwives	Enhanced midwife-doctor communication, confidence building for junior staff, and structured escalation protocols.
Technology adoption and implementation		
5	Training and digital confidence	Digital skills vary; success linked to training champions, mandatory sessions, and understanding tool rationale.
6	Ease of use	Intuitive design, straightforward interface, easy demographic input, and successful co-development outcomes.
7	Technology infrastructure barriers	Internet connectivity, Wi-Fi issues, device charging, CTG^b^ compatibility, and hardware availability.
8	Digital overload and change management	Too many tools and software can overwhelm; resistance to change needs addressing through buy-in engagement strategies.
Patient safety and decision-making		
9	Decision-making safety net	“Co-pilot” for clinical support, especially valuable in gray areas of CTG interpretation and safety net for junior staff.
10	Risk communication and intervention requests	Support for informed patient counseling with individualized risk information and transparent communication strategies. Concerns about sharing risk information could increase patient requests for interventions.
11	Clinical autonomy and professional responsibility	Tool should support and not replace clinical judgment; concerns around medico-legal risk and deskilling.
12	System-wide implementation and organizational adoption	Requires strong implementation planning, organizational readiness, and consistent departmental adoption and unified protocols.

a IUGR: intrauterine growth restriction.

b CTG: cardiotocography.

#### Clinical Integration and Workflow Domain

Across all sites, participants described the Fit4Labour tool as a valuable screening and triage aid, with color-coded risk stratification enabling rapid and intuitive prioritization. One Oxford doctor explained that the colors “acts as a powerful shortcut… one of the fastest and easiest ways to express risk.”

The tool was also seen as an effective escalation support mechanism, particularly for junior midwives who valued having objective evidence when calling senior staff: “You feel more confident having documentation that says you need to seek urgent medical advice” (Midwife, Oxford). Similarly, a Buckinghamshire midwife highlighted that the outputs facilitated communication by noting that *“*the Fit4Labour tool is one language…It helps put into words when you are not happy with a trace.”

However, limitations were also noted, including an incomplete risk factor profile, such as the absence of reduced fetal movements, small-for-gestational-age, previous stillbirth, and antepartum hemorrhage. Clinicians felt that these omissions could undermine accuracy and provide false reassurance, with one Oxford doctor commenting that “missing risk factors gives an impression of being very accurate and specific but may not be if you don’t input certain combinations.” Views on workflow integration varied, with some suggesting that 60-minute reports were impractical in fast-turnaround triage but potentially feasible in induction bays or antenatal wards.

#### Technology Adoption and Implementation Domain

Ease of use was widely recognized by all participants and was commonly described as being “very intuitive and straightforward.” A midwife at Oxford noted its accessibility even when fatigued, explaining that “it works for tired brains.” Some participants with visual impairments or neurodivergent profiles, including those who were color-blind or dyslexic, commented positively on accessibility, with one noting, “I can read it really clearly, I’m dyslexic.”

Nonetheless, a common theme was that structured training was essential given variation in digital literacy. A Birmingham doctor observed that “people that struggle with technology will still struggle because their confidence with IT is low *…* it’s not that this is difficult to use.” Suggested strategies included champion-led training and mandatory education programs. “People are always anxious with new technology, it needs mandatory training to use it properly, more reassurance to not miss things before using it” (Oxford Midwife).

Perceived implementation challenges extended to both infrastructure and organizational culture. Participants raised concerns about digital overload and imposed software changes in the NHS together with the need for senior buy-in, with one participant in Birmingham noting that “nobody likes change… you’ll need a lot of buy-in from senior people... it’ll snowball, just like iPhones when they came in..” Another common topic was infrastructure constraints, such as poor Wi-Fi, delays in IT support, missing chargers, and limited hardware, which was cited in Birmingham (“Wi-Fi doesn’t work, and you can’t see the CTGs*”*). In contrast, Buckinghamshire participants reported implementation would likely be easier as the hospital was already paperless.

#### Patient Safety and Decision-Making Domain

A prominent theme was the “co-pilot in a plane” analogy, first raised at Oxford, framing the tool as a supportive safety net rather than a replacement for clinical judgment, with a Birmingham doctor emphasizing that *“*the physician still has the ultimate decision-making.” The Fit4Labour tool was seen as particularly reassuring for junior staff and when presented with complex or ambiguous cases.

Participants valued the Fit4Labour tool to facilitate risk communication with patients, providing a “common language” for explaining probabilities and counseling women requesting care outside guidelines: “We are not informing patients of their real risks because we don’t know, but this is what Fit4Labour can bring” (Doctor, Oxford). Some clinicians cautioned that transparent risk data might increase cesarian requests “for the price of honest communication of risk,” while others felt it would reduce paternalism and enhance women’s autonomy. Buckinghamshire participants emphasized its role in countering misinformation, particularly the perception that cesarian birth is inherently safer: “Patients think c-section is the safer option, often misled by social media; the tool can help address this and decrease requests.”

Concerns regarding over-reliance and deskilling were also raised, with one participant warning that “it can deskill people… you will take shortcuts after a while.*” Moreover,* participants highlighted the need for clear protocols to ensure the tool complements rather than replaces expertise. Perspectives evolved across sites, shifting from early skepticism about being “told what to do by a machine” to recognition of the tool as “an important piece of the jigsaw.*”* Birmingham participants stressed balanced integration, underscoring that “your clinical judgment is always going to be important—see the patient beyond the technology.” They also noted potential to rebuild trust in maternity care, with one participant observing that “maternity is in the public eye because of serious incidents and poor care. Social media doesn’t help. Trust is broken. Fit4Labour can decrease this.”

### Mixed Methods Integration: Joint Display Analysis

Integration of quantitative and qualitative findings revealed strong convergence, with both complementary and contrasting insights (Table 4).

Convergence (direct alignment) was evident as high SUS and SEQ scores aligned with qualitative reports of intuitive design and ease of use, while completion times paralleled clinicians’ descriptions of smoother workflows, which may translate to more efficient practice.

Complementarity (mutual enhancement) also emerged, with quantitative improvements in task completion times complementing qualitative concerns about resource constraints and highlighting both usability and systemic challenges. Quantitative differences between midwives and doctors were minor, but differences became evident for qualitative themes around escalation support and risk communication.

Dissonance (contrasting perspectives) appeared in 2 key areas: while learnability scores were high, participants still requested structured training, indicating that initial perceived ease of use of the Fit4Labour tool does not eliminate the need for formal instruction regarding its clinical application. Similarly, strong usability ratings contrasted with concerns about incomplete or “missing” risk factor coverage—highlighting that usability alone does not guarantee clinical comprehensiveness and further understanding of the limitations of the tool is needed.

**Table 4.** Mixed methods integration summary: convergence, complementarity, and dissonance across findings. Joint display illustrating alignment between quantitative and qualitative results, highlighting usability, workflow efficiency, and training needs.

Integration pattern and key findings	Evidence
Convergence	
Excellent usability	Validation phase SUS^a^ scores (mean 85.8, SD10.2) independently met the "excellent" threshold and aligns with qualitative feedback stating both “very intuitive” and “easy to use.”
Co-development progression	SUS scores across co-development versions reflect iterative refinement reaching the excellent threshold by v3.0, consistent with participants’ growing familiarity with the tool’s ’co-pilot’ function.
Advanced feature complexity	More complex scenarios (eg, 4) had lowest scores (mean 5.7, SD 1.0), which aligns with concerns regarding the need for “structured and mandatory training.”
Complementarity	
Workflow integration	Within the validation phase, task completion times of 9.8 minutes were broadly feasible.
Performance predictability	Narrow SD (1.8 min) within the validation phase supported workflow consistency needs for ’rapid turnover in triage.
Professional group needs	Quantitative differences between midwives and doctors complemented qualitative emphasis on escalation support.
Infrastructure feasibility	Fast completion times (9.8 min) supported feasibility despite “Wi-Fi,” “charging,” and “compatibility” concerns.
Dissonance	
Learning versus training	High learnability scores (89.6%) contrasted with participants' expressed need for extensive training, reflecting the complexity of using the tool in clinical practice
Performance versus comprehensiveness	Excellent usability scores contrasted with concerns regarding “limited risk factors profile.”

a SUS: System Usability Scale.

## Discussion

### Main Findings

This multicenter mixed methods study demonstrated that the Fit4Labour tool developed through intensive co-design at a single NHS hospital independently met the “excellent” usability threshold (SUS >80) in simulated testing conditions at both validation sites, with consistent task completion times across 2 hospitals with different organizational contexts and digital maturity. Birmingham midwives reported lower usability scores (mean 76.3, SD 11.4), though still within the ’good’ range. This likely reflects the more critical evaluation standards based on their extensive clinical experience with existing triage systems, which strengthens the robustness of our validation findings.

Qualitative data reinforced these findings: participants described the Fit4Labour as a helpful triage and escalation tool, particularly for junior staff and as a shared “one language” for risk communication across professional boundaries. The spontaneous “co-pilot” analogy indicated that clinicians viewed the tool as supportive rather than substitutive, preserving autonomy while easing cognitive load. This preference for augmentation over automation mirrors findings from emergency triage settings, where professionals and patients alike favor hybrid digital-human approaches over fully automated solutions [44]. Although midwives and doctors have distinct roles, both groups reported that the interface met their needs, suggesting that a single design can accommodate diverse professional requirements in simulated testing conditions.

Clinicians also identified areas for improvement, including incomplete risk factor profile coverage, infrastructure barriers that may complicate seamless integration, and the need for structured training to support consistent use.

### Comparison With Prior Work

Earlier evaluations of computerized cardiotocography systems in randomized controlled trials have shown limited benefit, with no clear improvements in neonatal outcomes or reductions in unnecessary interventions [45,46]. Systematic reviews suggest that for decision-support tools to make a real difference, they must be interpretable, reproducible, and trusted by clinicians, rather than simply replicating the same inconsistencies seen in human interpretation [45,47,48]. Fernandes et al [49]*,* in a systematic review of intelligent clinical decision support systems for emergency triage, found that many such systems lack a formal implementation phase and that validated systems consistently improved clinician decision-making; this study directly addresses this gap by combining algorithmic development with structured multicenter usability evaluation.

The Fit4Labour tool represents a promising evolution in this field. Unlike earlier systems based purely on human [Bibr R49]cardiotocography pattern recognition, it is a prognostic model trained on data from a large, multicenter clinical database, allowing it to produce standardized, evidence-based risk scores [32,33]. By providing clear, data-driven outputs, clinicians perceived the tool as having the potential to reduce variation in cardiotocography interpretation and to support less experienced staff, while complementing rather than replacing senior clinical judgment, perceptions that align with the tool’s design intent but require prospective clinical evaluation to confirm.

These advantages are particularly relevant in the current maternity safety landscape. The UK Healthcare Safety Investigation Branch recently highlighted how unpredictable workloads and a constant need to juggle tasks in labor wards can compromise situational awareness and delay recognition of fetal compromise [50]. Participants in this study felt that the Fit4Labour tool could help maintain an overview of the full clinical picture, reduce cognitive load, and minimize the risk of overlooking important patient information. By alerting users when data is missing or incomplete, the tool also aligns with human-factors principles of error-tolerant design [51].

Similarly, national inquiries such as Ockenden and MBRRACE have underscored the role of poor communication and delayed escalation in adverse outcomes [29,30]. These findings resonate with observations from our study, where participants described using vague, non-specific expressions over the phone (eg, “I’m not happy with this trace”) when escalating concerns about cardiotocography traces. Clinicians suggested that a numeric risk score could help reduce subjectivity in intrapartum communication, though this needs to be confirmed in adequately powered prospective studies.

Previous evaluations of computerized cardiotocography systems have reported variable user acceptance due to unclear risk calculations and scoring methods [45,52,53]. In contrast, participants found the Fit4Labour tool intuitive and transparent, which likely contributed to its positive usability scores. They also expressed interest in greater clarity regarding how individual maternal and fetal factors influence the overall risk score, including the relative weight of each contributing variable. Showing how these factors, together with cardiotocography interpretation and population data, feed into the score could help clinicians better understand and trust the tool’s output, rather than viewing it as a “black box.”

Finally, participants valued the unified interface that supported both midwives and doctors. Our findings indicate that a shared, role-sensitive interface can accommodate diverse professional needs while promoting interprofessional communication. This aligns with human-factors literature advocating shared mental models to bridge doctor–midwife silos, reduce fears of hierarchical barriers when escalating, and ensure complete, consistent communication in high-risk settings [54,55]. The slightly lower scores observed among senior midwives at Birmingham are consistent with evidence from emergency department settings, where senior physicians have been shown to report greater frustration with health IT systems than residents, attributed to higher expectations shaped by prior workflows. [56]

### Human Factors Innovation: Cognitive Support and Communication

Intrapartum care involves complex decisions under uncertainty, where cognitive load, fatigue, and time pressure can compromise situational awareness. Participants described the Fit4Labour tool as “working for tired brains,” highlighting its perceived value under conditions of fatigue, multitasking, and workload pressures, particularly relevant in maternity care. The design also appeared to support progressive disclosure: junior staff could rely on simple, color-coded outputs for rapid decision-making, while senior clinicians valued the ability to review detailed cardiotocography metrics and contributing risk factors. This layering helped ensure that transparency and autonomy were maintained without overloading users. In addition, when information was incomplete or omitted, the tool prompted users to add missing data, helping to reduce errors.

### Clinical Integration and Implementation Considerations

In our analysis of over 147,000 births, the Fit4Labour algorithm identified about 1 in 3 babies who later showed signs of severe compromise, using only the first 20 minutes of cardiotocography at the start of labor [34]. Many of these babies were not recognized until 8‐10 hours later, when emergency intervention was needed, suggesting that harm experienced during the birth process might be curtailed or even prevented. Although these results come from retrospective cohort analysis rather than real-time use, participants suggested that making risk visible, readily available, and quantifiable could support earlier and more consistent decision-making, especially when early cardiotocography changes are subtle. With maternity-related litigation in the UK now exceeding £37 billion (more than the annual cost of running maternity services), there is an urgent need for preventive, evidence-based innovations that reduce avoidable harm and help rebuild trust in care [57, 58].

Participants across all sites emphasized that structured clinician education remains essential for effective and consistent implementation. Training should go beyond button-clicking or navigation (interface use), focusing on supporting decisions and helping clinicians interpret risk, and communicating effectively with women. This approach ensures that the Fit4Labour *tool* outputs are translated into safe, evidence-based care rather than reducing decision-making to a “tick-box” exercise.

Interestingly, over nearly 2 and a half years of sessions, a noticeable shift in participants’ views emerged, reflecting the broader uptake of digital technologies and AI in health care, with early anxieties about “being told what to do by a machine” evolving into recognition of a novel digital tool as a potentially “important piece of the puzzle” that enhances, rather than constrains, professional autonomy. This gradual shift also highlights increasing confidence in technology and the potential of digital tools to support decision-making and improve escalation pathways [59]. This trajectory is consistent with Longoni et al.’s framework, in which resistance to medical artificial intelligence is primarily driven by “uniqueness neglect,” the concern that algorithms cannot account for individual patient characteristics. Fit4Labour tool’s transparent, interpretable outputs and preservation of clinical autonomy directly address this concern. [60]

Organizational readiness is likely to play a pivotal role in adoption. Digitally mature sites, such as Buckinghamshire, noted that the Fit4Labour tool could be easily integrated into existing workflows, while others cited unreliable Wi-Fi, limited computer access, poor and delayed IT support, and competing software as major barriers. These findings echo ‘Consolidated Framework for Implementation Research (CFIR) v2.0,’ emphasizing that infrastructure, culture, and implementation support, through training, local champions, and change management, are as important as usability for successful adoption [61].

Equity considerations also featured prominently in group discussions. Clinicians acknowledged the steps taken during co-development to promote inclusiveness in the user interface. These included design choices tested for accessibility among color-blind and dyslexic users, adaptations for staff wearing gloves when using touch tablets, such as larger buttons, and adjustments to font and button sizes to accommodate differences in visual acuity and hand size. However, participants also highlighted areas for further improvement. The current version is available only in English, which may create barriers for clinicians when explaining risk to non–English-speaking patients. Participants noted that using an interpreter or relying on a family member for translation, particularly over the phone, can make it more difficult to use the tool effectively in real time. Cultural factors may also influence how risk is communicated and perceived, underscoring the need for continued research and iterative refinement. Finally, participants noted that ethnic minority groups were not specifically represented and are likely underrepresented in the dataset used to refine the Fit4Labour algorithm, which could limit its generalizability. Ensuring inclusive design and representative data will be essential to prevent the reinforcement of existing inequities in maternity care [62].

### Practical Implication for Digital Health Tools Development

Digital health projects often allocate significant resources to local customization (often between 15%‐20% of implementation budgets) [63-65]. In this study, the Fit4Labour tool maintained high usability across 3 hospitals with different organizational contexts and IT infrastructures when tested in the same version. This observation raises a practical question of whether intensive co-development at the outset might reduce the need for postdeployment customization. However, establishing this would require prospective studies comparing implementation costs and outcomes for tools developed with versus without intensive participatory design.

### Future Research

Our team will conduct a regulated clinical evaluation of the Fit4Labour tool across multiple NHS sites to assess real-world safety, usability, and workflow integration, in line with Medicines and Healthcare products Regulatory Agency requirements for medical device approval [66]. This study will focus on outcomes such as escalation timeliness, reliability of communication, handover quality, and patient safety indicators.

We will also examine factors influencing sustained use and implementation, including the impact of structured training, local clinical champions, and integration into existing hospital systems. In parallel, our team will build on early health economic modeling to assess whether the usability patterns observed in this study translate into measurable cost savings and better clinical outcomes.

Beyond our own work, external validation in non-NHS health care systems is an important next step to determine whether the tool’s core design principles transfer to other diverse health care contexts.

Finally, we will continue to engage directly with women, families, and PPI panels to explore how different ways of presenting risk information affect perception, autonomy, and trust. Embedding patient perspectives throughout these evaluations will help ensure that digital tools strengthen shared decision-making and enhance both safety and the experience of care.

### Strengths and Limitations

This study has several strengths. The multicenter design, spanning 3 NHS hospitals with different organizational contexts and levels of digital maturity, provided a robust test of both usability and scalability across diverse settings. The mixed methods approach enabled convergence of quantitative usability metrics with qualitative insights into workflow, communication, and professional practice. Importantly, the locked-version (v4.0) validation offered a more rigorous test of scalability across diverse settings than is usually attempted in health care technology evaluations.

Our study also has important limitations. Usability testing was conducted in structured environments rather than during real-time clinical care, which may have increased observed task efficiency. However, as all sites used the same testing protocol, the relative comparisons between validation sites remain valid, even if absolute values might differ in clinical practice. Recruitment through departmental managers could have favored digitally confident clinicians, although efforts were made to capture a range of roles and experience. The involvement of members of the development team in evaluation may also have introduced bias, but this was mitigated by standardized protocols and independent analysis. The relatively small sample size (n=26) is consistent with Nielsen’s guidance for usability studies and similar mixed methods implementation studies. No inferential statistical testing was performed, which is consistent with the exploratory descriptive aims of this work. Moreover, phase 1 and phase 2 differed simultaneously in tool version, participants, and clinical site, making between-phase inferential comparison inappropriate. Finally, the study was confined to NHS hospitals in the United Kingdom, which limits direct generalizability to health care systems with different funding models, workforce structures, technological infrastructures, or cultural norms. However, the 3 sites were selected to capture meaningful variation in organizational context and digital maturity, and the human-factors frameworks and usability instruments applied are internationally validated. The core challenges addressed, variable cardiotocography interpretation, escalation delays, and risk communication, are not unique to the NHS and have been documented in maternity systems internationally. Future evaluation in non-NHS settings will be essential to establish broader applicability.

### Conclusions

This study demonstrates that systematic co-development, grounded in human-factors principles, can produce clinician-facing decision-support tools that independently meet established usability benchmarks across NHS hospitals with different organizational contexts and digital maturity. The locked version (v4.0) showed high usability at both validation sites (SUS mean 80.7, SD 10.8 Birmingham vs SUS mean 90.8, SD 7.2 Buckinghamshire). Clinicians described the Fit4Labour tool as a supportive, like a “co-pilot” rather than a replacement for clinical judgment, and that it provided a shared language for risk communication with potential to strengthen confidence in escalation decisions. Other factors, such as organizational readiness, structured training, and adequate infrastructure support, will be important enablers of adoption. Whether usability in simulated settings translates to improved clinical outcomes in real-world practice, and whether these findings generalize beyond NHS settings, requires prospective evaluation.

## Supplementary material

10.2196/87589 Multimedia Appendix 1 Video demonstration of the Fit4Labour tool showing clinical workflow and user interface.

## Abbreviations

BHT: Buckinghamshire Healthcare NHS Trust
BWC: Birmingham Women’s and Children’s NHS Foundation Trust
NHS: National Health Service
NIHR: National Institute for Health and Care Research
OUH: Oxford University Hospitals NHS Foundation Trust
PPI: patient and public involvement
SEIPS: Systems Engineering Initiative for Patient Safety
SEQ: Single Ease Question
SUS: System Usability Scale
UK: United Kingdom
