# Supplementary material for: Multicenter Usability Evaluation and Co-Development of a Digital Decision-Support Tool for Labor Triage: Mixed Methods Study
Source: JMIR Hum Factors. 2026 Jul 23;13:e87589. doi: 10.2196/87589 (PMC13395436; doi:10.2196/87589)
Supplement: Multimedia Appendix 1 [file humanfactors-v13-e87589-s001.docx]

**SUPPLEMENTARY MATERIAL**

**Multicentre Usability Evaluation and Co-Development of a Digital Decision-Support Tool for Labour Triage: Mixed-Methods Study**

**Table of contents**

[**List of Abbreviations** 2](#_Toc229491844)

[**Table S1**. Summary of the scenarios tested in the usability session. 3](#_Toc229491845)

[**Table S2.** Participant characteristics across sites. 4](#_Toc229491846)

[**Table S3.** Summary design modifications during the study. 5](#_Toc229491847)

**[Table S4.](#_Toc229491848)**  [Task completion times analysis by study phase and site 10](#_Toc229491848)

[**Figure S1.** Usability Session Structure. 11](#_Toc229491849)

[**Figure S2.** The 10-item System Usability Scale (SUS) questionnaire used during usability sessions. 12](#_Toc229491850)

[**Figure S3.** Single Ease Question Score (SEQ) used during usability sessions. 13](#_Toc229491851)

[**Supplementary Analysis 1: Detailed Theme-by-Theme Analysis** 14](#_Toc229491852)

[CLINICAL INTEGRATION AND WORKFLOW DOMAIN 14](#_Toc229491853)

[TECHNOLOGY ADOPTION AND IMPLEMENTATION DOMAIN 18](#_Toc229491854)

[PATIENT SAFETY AND DECISION-MAKING DOMAIN 21](#_Toc229491855)

[**Supplementary Analysis 2: Site-Specific Thematic Analysis** 26](#_Toc229491856)

[Oxford University Hospitals (Co-development Site) 26](#_Toc229491857)

[Birmingham Women's and Children's Trust (First Validation Site) 27](#_Toc229491858)

[Buckinghamshire Hospital (Second Validation Site) 27](#_Toc229491859)

[**Supplementary Analysis 3: Professional Group Stratified Analysis** 28](#_Toc229491860)

[**MIDWIVES' PERSPECTIVES** 28](#_Toc229491861)

[**DOCTORS' PERSPECTIVES** 29](#_Toc229491862)

[**PROFESSIONAL GROUP CONVERGENCE** 29](#_Toc229491863)

[**Multimedia Appendix 1.** (Video) 31](#_Toc229491864)

# **List of Abbreviations**

**APH** – Antepartum haemorrhage
**BMI** – Body mass index
**CTG** – Cardiotocography
**DC** – Deceleration capacity
**FAQ** – Frequently asked questions
**GA** – Gestational age
**IUGR** – Intrauterine growth restriction
**MAU** – Maternity Assessment Unit
**NHS** – National Health Service
**PROM** – Premature rupture of membranes
**SROM** – Spontaneous rupture of membranes
**STV** – Short-term variation
**SUS** – System Usability Scale
**SEQ** – Single Ease Question

# **Table S1**. Summary of the scenarios tested in the usability session.

| **SCENARIO 1** | **SCENARIO 2** | **SCENARIO 3** | **SCENARIO 4** |
| --- | --- | --- | --- |
| - Add risk factors - Read the 60-minute report - Save the record | - Add risk factors - Add a Note - Confirm note was saved - Read the 60-minute report - Save the record | - Add risk factors - Read the 20-minute report - Add a new Risk factor and update - Read the 60-minute report - Save the record | - Add risk factors - Read the 60-minute report. - Find Advance Features. - Record STV value. - Save the record |
| **Low complexity High Complexity** | | | |

A standardised task-based evaluation framework was applied across all three NHS sites to assess user performance, task completion efficiency, and scenario-specific usability barriers. Each scenario builds in complexity from basic functionality (Scenario 1) to advanced feature utilisation (Scenario 4), enabling systematic assessment of learning curve progression and identification of usability challenges across different levels of tool engagement.

# **Table S2.** Participant characteristics across sites.

| **Site / Phase** | **Total n** | **Midwives**  **n (%)** | **Doctors**  **n (%)** | **Consultant**  **(doctors)**  **n (%)** | **Trainee**  **(doctors)**  **n (%)** | **Female participants, n (%)** |  |
| --- | --- | --- | --- | --- | --- | --- | --- |
| Oxford  (Phase 1) | 12 | 7 (58) | 5 (42) | 3 (60) | 2 (40) | 10 (83) |  |
| Birmingham  (Phase 2) | 8 | 5 (62) | 3 (38) | 2 (67) | 1 (33) | 7 (88) |  |
| Buckinghamshire  (Phase 2) | 6 | 3 (50) | 3 (50) | 1 (33) | 2 (67) | 5 (83) |  |
| **Total** | **26** | **15 (58)** | **11 (42)** | **6 (55)** | **5 (45)** | **22 (85)** |  |

Twenty-six healthcare professionals participated across three sites: 15 midwives (58%) and 11 doctors (42%; 6 consultants, 5 trainees). Overall, 85% of participants were female. **Note.** One Birmingham midwife observed the scenarios only and participated solely in the focus groups, resulting in n = 26 total participants but n = 25 for scenario assessments.

# **Table S3.** Summary design modifications during the study.

| **Version Transition** | **Issue Identified** | **Specific Problem** | **Design Modification Implemented** | **Impact Assessment** |
| --- | --- | --- | --- | --- |
| **VERSION 1.0 → 2.0**  **Post-Initial Midwives Session (December 2022)** | **Button Selection State** | "Auto select buttons and colours confusing. Unsure to know if the button was already selected or not with the colour scheme. When it came up it was greyed out, was unsure if what I selected was selected" | Redesigned button colour scheme: Selected buttons now display distinct visual state with clear colour differentiation from unselected state | Eliminated selection confusion and improved visual feedback |
|  | **Keyboard Functionality** | "Keyboard at input data moment, the keyboard calendar kept popping up and wouldn't leave (got in the way/frustrating)" | Implemented automatic keyboard dismissal after input completion; fixed calendar popup persistence issue | Reduced input frustration; improved data entry efficiency |
|  | **Decimal Input Functionality** | "The decimal point for temperature didn't work" | Fixed decimal point input functionality for numerical fields, particularly temperature entry | Enabled accurate temperature recording |
|  | **Advanced Analysis Display** | "There are no numerical features for STV (on advanced analysis), the first dot covers the numbers (on the side)" | Repositioned visual elements in advanced analysis to prevent overlap; improved numerical display visibility | Enhanced readability of STV measurements |
|  | **Suggestion of FAQ button** | "If I’m working a night shift alone after not using this for a while, I’d need an easy way to review definitions and criteria." | Added a FAQ with all the questions suggested and definitions. | Clearer clinical guidance on optimal use of the tool |
|  | **Advanced Analysis Button Size** | “Is difficult to see where the advance analysis button is, I couldn’t find it straight away” | Adjusted button sizing for improved touch interface usability in clinical environment | Enhanced usability for clinical setting usage; improved touch interface responsiveness |
|  | **Notes Functionality Discovery** | "No one noted that they could add a note via tapping on the notes or the CTG, no way to know unless you have prior knowledge" | Added visual indicators and instructional text to highlight note-taking functionality | Improved feature awareness and utilisation |
|  | **Due Date Entry Visibility** | "Missed the due date entry, because already populated with today's date. Maybe be easier to notice if populated with 00-00-00" | Changed default date field display to placeholder format (00-00-00) instead of auto-populated current date | Increased user attention to date field requirements |
|  | **Established Labour Definition Clarity** | “I am confused with the definition being used for 'established labour and non-established labour, it varies a lot” | Information added to FAQ section and added to future training package | Improved clinical guidance for labour assessment; reduced ambiguity in clinical decision-making |
|  | **Report Timing Clarity** | "Didn't find the 20-minute report obvious once ready to be read" | Enhanced visual prominence of 20-minute report notification; added clear timing indicators | Improved awareness of available reports |
|  | **Bad Outcome Definition Understanding** | "Not sure what the definition of a 'bad outcome' is” | Added an explanation to FAQ Help questions. Also, team will take this in consideration for training prior to implementation | Enhanced clinical usability; clearer understanding of what the risk score means |
|  | **Advanced Analysis Report Simplification** | “I found the 60-minute report confusing with a lot on unnecessary information and wanting clearer urgency indicators | Simplified the 60-minute advanced analysis report format, reduced unnecessary complexity, and improved the clarity of urgency indicators. | Enhanced clinical usability; clearer guidance on patient management priorities |
|  | **Risk Factor Coverage** | "There were not enough risk factors to choose from" | Ongoing work to expand risk factors, such as BMI, IUGR, PROM, etc | Enhanced clinical comprehensiveness within model limitations |
| **VERSION 2.0 → 3.0**  **Post-Second Midwives Session (February 2023)** | **Report Navigation** | "The task asking them to navigate back to the 20-minute report past 60 minutes isn't very obvious" | Implemented breadcrumb navigation system; added clear pathways between different report timepoints | Simplified report access and timeline navigation |
|  | **Risk Factor Organisation** | General feedback about risk factor categorisation on the interface | Reorganised risk factors into logical clinical categories: maternal factors, fetal factors, and labour factors | Improved clinical relevance and input efficiency |
|  | **Risk Factor Coverage** | "There were not enough risk factors to choose from" and "With the undiagnosed IUGR, explained she put into the notes but wanted to put it as a risk factor" | Ongoing work to expand risk factors, such as BMI, IUGR, PROM, etc.. | Enhanced clinical comprehensiveness within model limitations |
|  | **Risk Visualisation** | "Felt it took a while to see what part was highlighted on report (letters) and her eyes were drawn to the background risk. Better if came up displayed simply as 'high risk'" | Redesigned risk infographic with clearer visual hierarchy; added prominent risk category labels (high/medium/low) at top of display | Improved rapid risk assessment and visual scanning |
|  | **CTG Analysis Transparency** | "Can we have a way of adding 'CTG analysis' as a contributing factor when the CTG is actually pulling it to amber" | Added specific CTG parameters to contributing factors display: Baseline >150 or <110, DC average <1.5 or >5.8, STV average <5 | Enhanced transparency in risk assessment; clinicians can see which CTG features drive risk classification |
|  | **Clinical Threshold Refinement** | "DC was low 1.56 (limit is 1.5). I am not sure I would describe the trace as borderline non-reactive" | Lowered DC thresholds: Borderline from 2.0 to 1.5, non-reactive from 1.2 to 1.1 to reduce amber cases | Refined clinical classification accuracy; reduced false positive amber classifications |
|  | **User Support Documentation** | "Add if fetal movements are important for the algorithm answer is no" | Planned addition of fetal movement information to FAQ section | Improved user understanding of algorithm parameters and limitations |
|  | **Due Date Entry Field Visibility** | “I have missed entering the due date correctly, as I have a cold and I am dyslexic, so may have missed” | Changed to placeholder format (00-00-00), unable to move on without correctly completing it | Highlighted both the general improvement and the accessibility benefit for users with dyslexia |
|  | **Risk Infographic Clarity** | The quote about the infographic being unclear and taking time to understand the risk level | Redesigned risk infographic with clearer visual hierarchy; added prominent risk category labels (high/medium/low) at top of display | Improved rapid risk assessment and visual scanning; reduced cognitive load for busy clinicians |
|  | **Patient communication guideline** | "Could you add a refresher on patient communication to the report? It would be helpful to know how to share this information clearly." | Included a new section in the report on communicating risk categories (low, medium, high) to patients. | Improved user confidence in communicating risk to patients |
| **VERSION 3.0 → 4.0**  **Post-Doctors Sessions (November 2023 - January 2024)** | **Information Display Hierarchy** | "I want to understand which risk factors carry the greatest weight in raising the risk score." | Optimised information display hierarchy to match clinical decision-making priorities | Improved clinical utility and scanning efficiency |
|  | **Advanced Analysis Button Size** | "The size of advance analysis buttons" | Adjusted button sizing for improved touch interface usability in clinical environment | Improved touch interface responsiveness |
|  | **Report Accuracy Communication** | "Can we add a message about how accurate the 20-minute report is? for people to be aware that 60-minute trace is more accurate" | Addition of accuracy disclaimer to the FAQ section | Informed clinical decision-making; appropriate use of preliminary vs full reports |
|  | **Risk Detection Sensitivity** | "Scenario 4 on usability session has 2 prologued decels but says it is low risk" | Updated algorithm and improve deceleration detection | Enhanced identification of concerning patterns |
|  | **Gestational Age Input Validation** | "If the Gestation Age that is added is too long ago the app crashes and becomes red report" | Redesigned so that if the GA exceeds 44 weeks, the user is notified to review the due date, as this is likely an error. | Improved system stability; prevented crashes from data entry errors |
|  | **Button Instruction Clarity** | "Buttons with instructions written on them EDIT NOTES, SAVE, Edit risk factors. It is better for buttons to explain their action before pressing them.” | Add descriptive labels to buttons clearly explaining their function before user interaction | Enhanced user confidence; reduced uncertainty about button functions |
|  | **Advanced Analysis Button Accessibility** | "View Advance analysis difficult to press the advance analysis buttons" | Enlarge size of the buttons on the advance analysis interface | Improved touch interface usability; better accessibility for clinical environment |
|  | **Quick Notes Enhancement** | "Add prewritten sentences that people will write in the notes without taking too long typing (e.g.: patient in the toilet, patient very distressed, patient vomiting)" | Implementation of quick-select note options with common clinical observations | Enhanced documentation efficiency; reduced typing burden in busy clinical settings |
|  | **Risk Graphic Intuitive Design** | "Graphic numbers and size are confusing. The risk number doesn't correspond with the place on the bar. Not intuitive" | Redesign risk visualisation to ensure visual representation accurately matches numerical risk values | Improved risk comprehension; enhanced visual-numerical correspondence |
|  | **Report Timeline Identification** | "The report that you are reading is not clear if 20m or 60m. Can we add on the 20-minute report box on the top saying that it is the 20-minute report and on the 60m as well" | Add clear timeline indicators on both 20-minute and 60-minute report headers | Improved report identification; reduced confusion about report timing |
|  | **Advanced Analysis Button Labelling** | "Advance analysis button participants didn't see it as something to press to get all the information. Can we change the name to advance analysis summary" | Rename button to "Advance Analysis Summary" for clearer functionality indication | Enhanced feature discoverability; improved user understanding of button purpose |
|  | **Patient Data Persistence Issue** | "When you open the second usability patient, the details of previous patient were there. The GA and due date" | Error identified as issue specific to testing version and not in the live patient scenarios | Improved data integrity; prevented cross-patient data contamination |
|  | **Information Summary Feature** | "Can we have a button with the summary of the information entered? Like bullet points, e.g., Patient Risk factors: 38 weeks, temperature 38, induction, and meconium" | Consider implementation of summary overview button with key patient information display | Enhanced information overview; improved clinical decision-making support |
|  | **Visual Risk Status Indicators** | "Can the colour of the room change to red if the report is red? Where the columns are" | Implement colour-coded room status indicators matching risk assessment results | Enhanced visual risk identification; improved workflow integration and patient prioritisation |
|  | **Risk Factor Coverage** | "I wanted to add IUGR and it wasn’t there” | Ongoing work to expand risk factors, such as BMI, IUGR, PROM, etc.. | Enhanced clinical comprehensiveness within model limitations |

Iterative design improvements were implemented during the co-development phase at Oxford University Hospitals across four software versions. Final version 4.0 was subsequently tested at Birmingham Women's and Children's Trust and Buckinghamshire Healthcare Trust for validation. **BMI,** body mass index; **CTG**, cardiotocography; **DC**, deceleration capacity; **FAQ**, frequently asked questions; **GA,** gestational age; **IUGR,** intrauterine growth restriction; **PROM**, premature rupture of membranes; **STV**, short-term variation.

# **Table S4. Task completion times analysis by study phase and site**

| **Phase** | **Site** | **Professional Group** | **Participants**  **(n)** | **Version Tested** | **Mean Time (SD)** | **Range** | **Study Phase** |
| --- | --- | --- | --- | --- | --- | --- | --- |
| **Co-Development** | Oxford | Midwives | 7 | v1.0-2.0 | 11.6 (1.0) | 11-14 min | Phase 1 |
|  |  | Doctors | 5 | v3.0 | 12.2 (3.7) | 7-18 min | Phase 1 |
|  |  | **Co-development Total** | **12** | v1.0-3.0 | **11.8 (2.5)** | **7-18 min** | **Phase 1** |
| **Validation** | Birmingham | Midwives | 4 | v4.0 | 11.0 (1.0) | 10-12 min | Phase 2 |
|  |  | Doctors | 3 |  | 9.3 (1.7) | 7-11 min | Phase 2 |
|  |  | **Site Subtotal** | **7** |  | **10.3 (1.6)** | **7-12 min** | **Phase 2** |
|  | **Buckingham-shire** | Midwives | 3 | v4.0 | 9.0 (2.4) | 6-12 min | Phase 2 |
|  |  | Doctors | 3 |  | 9.3 (0.9) | 8-10 min | Phase 2 |
|  |  | **Site Subtotal** | **6** |  | **9.2 (1.9)** | **6-12 min** | **Phase 2** |
|  |  | **Validation Total** | **14** | v4.0 | **9.8 (1.8)** | **6-12 min** | **Phase 2** |
| **Overall Study** |  | **All participant**^†^ | **25** | **v1.0-4.0** | **(2.3)** | - 1. **min** | **Phase 2** |

Note^†^: One Birmingham midwife observed scenarios only and participated solely in focus groups, resulting in n=26 total


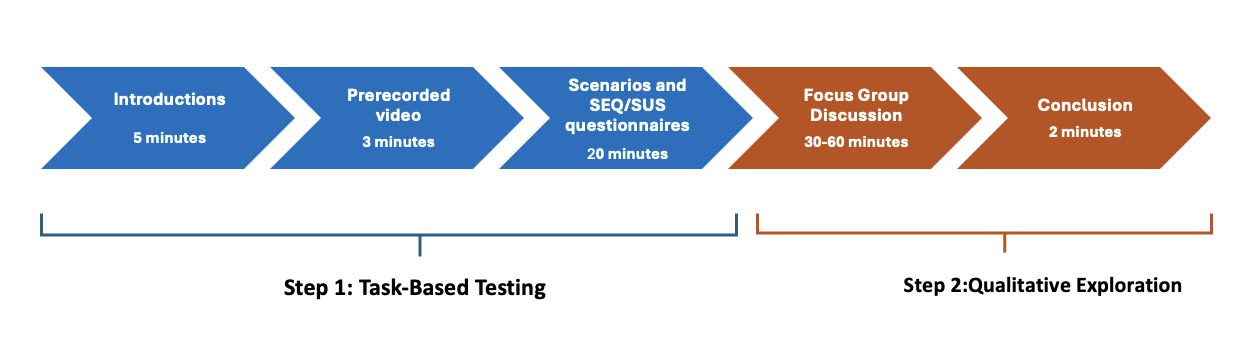


**Figure S1.** Usability Session Structure. Two-phase mixed-methods session protocol combining quantitative usability metrics with qualitative user experience exploration. Step 1 employed silent observation during task-based testing with standardised scenarios, while Step 2 utilised profession-stratified focus groups and individual interviews to capture comprehensive user perspectives on clinical integration, workflow implications, and implementation considerations.


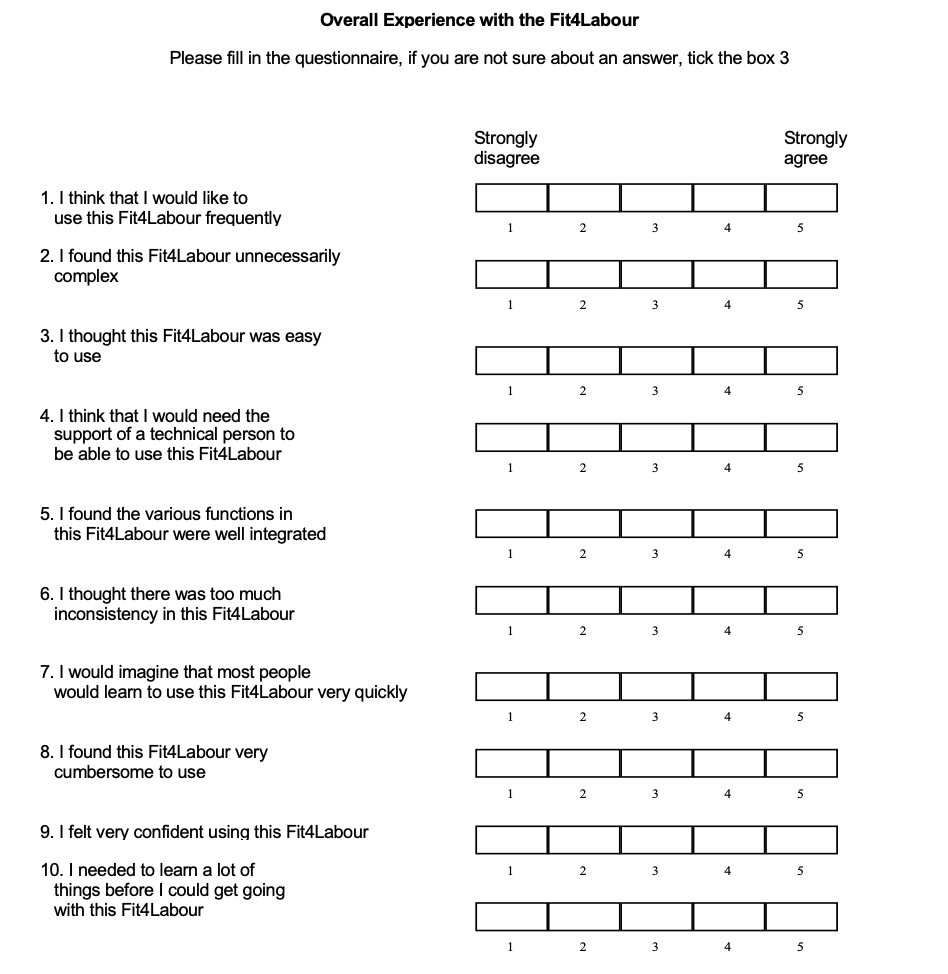


# **Figure S2.** The 10-item System Usability Scale (SUS) questionnaire used during usability sessions.


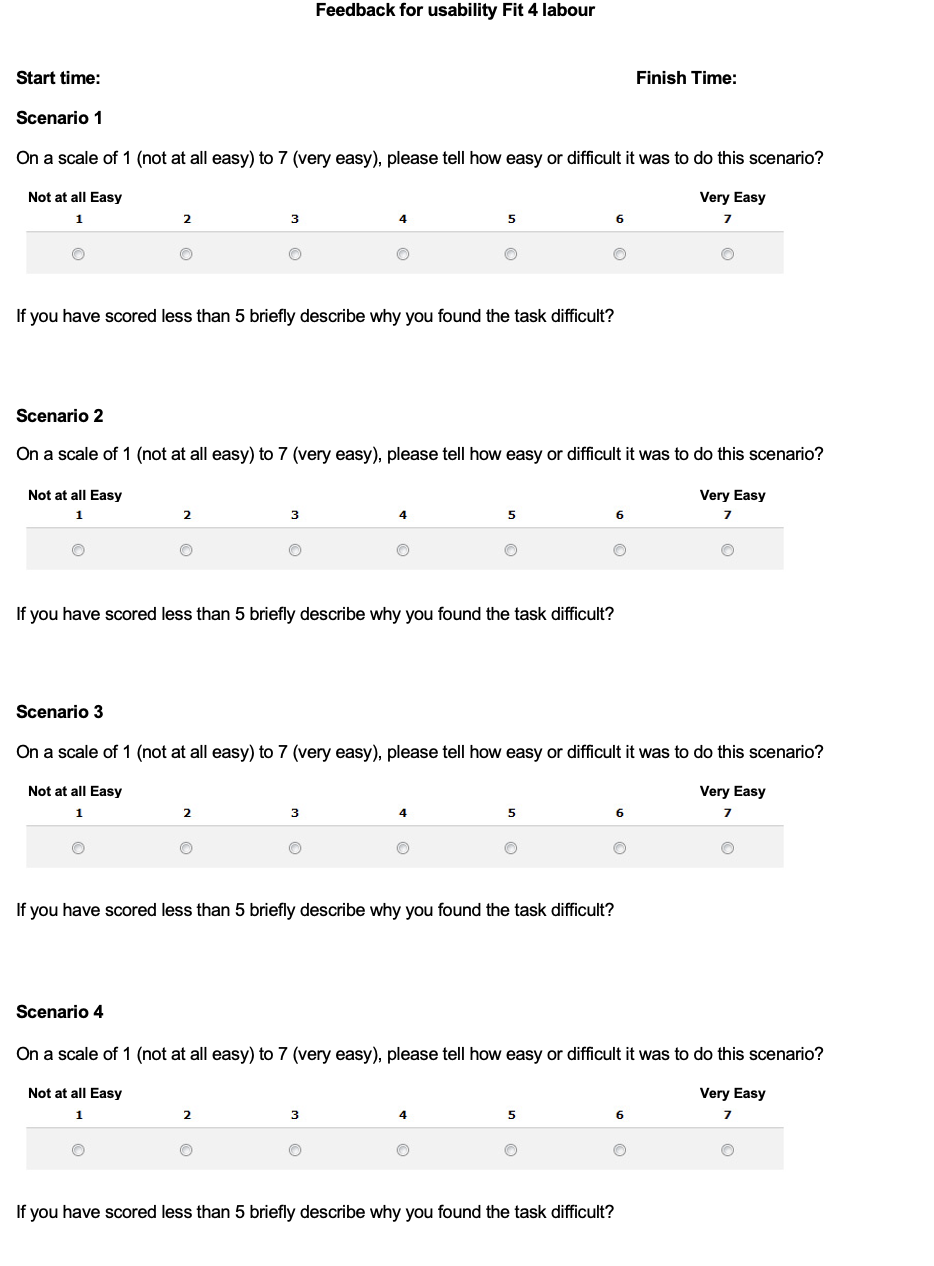


# **Figure S3.** Single Ease Question Score (SEQ) used during usability sessions.

# **Supplementary Analysis 1: Detailed Theme-by-Theme Analysis**

### CLINICAL INTEGRATION AND WORKFLOW DOMAIN

#### Theme 1: Risk Factors and Clinical Comprehensiveness

Participants across all sites consistently identified the need for more comprehensive risk factor coverage as a critical area for tool enhancement. The feedback demonstrated increasing clinical sophistication from Oxford through to Buckinghamshire, with validation sites providing detailed examples of missing clinical parameters that could impact risk assessment accuracy.

**Oxford baseline concerns:**

- "I was looking for the IUGR [intrauterine growth restriction] option on the risk factors and it wasn't there. Can we add more risk factors to the options?" (Oxford Midwife)
- "Missing risk factors... it gives an impression of being very accurate and specific... but may not be if you don't input certain combinations of risk factors." (Oxford Doctor)

**Birmingham validation feedback:**

- "There were only a few risk factors." (Birmingham Midwife)
- "So, someone does SROM [spontaneous rupture of membranes], and they've had a period of prolonged duration, and you've not got a fever. You would classify her on the *Fit4Labour* as low risk... but the fact that she's actually prolonged means that she is at risk of baby being not well." (Birmingham Doctor)

**Buckinghamshire** **comprehensive assessment:**

- "I suppose I was surprised, you know, we know this person's a smoker, there's a limited list of risk factors and things like, you know, APH [antepartum haemorrhage] or previous stillbirth isn't on there, reduced movements isn't on there... Your instinct is to write everything down, isn't it? All the risk factors... and then just be like go computer." (*Buckinghamshire* Midwife)
- "It needs to be used in a holistic and supportive way." (*Buckinghamshire* Midwife)

#### Theme 2: Timeline Duration and Workflow Integration

Participants provided nuanced feedback about timing options, with site-specific workflow considerations revealing resource allocation challenges.

**Oxford workflow insights:**

- "Favourite thing was the 20-minute report." (Oxford Midwife)
- "60 minutes is a long time in rapid turnover in MAU [Maternity Assessment Unit], patients need to be seen quicker to not block the space." (Oxford Midwife)
- "They need to be strapped for 1 hour is a lot for a labouring woman." (Oxford Midwife)

**Birmingham resource planning:**

- "Committing to doing it for everyone that is high risk that needs a CTG. Triage is not geared to support this to be for 1 hour - not enough rooms and midwives. Realistically it would be difficult." (consensus from multiple Birmingham Midwives)
- "Antenatal wards and induction bays having traces to 60 minutes I don't think is unreasonable and definitely is achievable. The main issue would be at triage as it is very busy." (Birmingham Midwives)

#### Theme 3: Screening and Triage Effectiveness

The tool's screening value was consistently recognised across all sites, with validation sites showing enhanced appreciation and specific applications.

**Oxford recognition:**

- "It is a great screening test." (Oxford Midwife)
- "It will be helpful for midwives' job as they are the gatekeepers to obstetrics...they make all the assessments, whether the women in labour or not, if needs a CTG or if a woman needs to come into the hospital." (Oxford Doctor)
- "Colour acts as a powerful shortcut: our brains process around 11 million pieces of information, so you need shortcuts, and colour is one of the fastest and easiest ways to express risk." (Oxford Doctor)
- "Colours speak easily to tired brains and patients in pain understand colours." (Oxford Doctor)

**Birmingham triage application:**

- "I feel it can be used nicely in triage. So, for the midwives? Yeah. To triage patients like green, red and amber... It will pick cases. So, they will come and say, oh, look, it's red. Would you want to come and see her immediately? So, this will help for some ladies not to wait" (Birmingham Doctor)
- "I love the colours, people love colours." (Birmingham Doctor)

**Buckinghamshire** **strategic implementation:**

- "They have so much to do, the senior midwives will feel safer for the junior midwives to have it." (*Buckinghamshire* Midwife)
- "I think the colours are helpful for people as like a visual aid of how urgently you need to sort out your patient." (*Buckinghamshire* Midwife)

#### Theme 4: Escalation Support for Midwives

While support for midwife-doctor communication was valued across all sites, concerns emerged about potential delayed escalation if staff waited for the 20-minute report in emergency situations.

**Oxford escalation confidence:**

- "I have issues escalating. If you haven't been qualified that long you will feel more confident in having a piece of paper that said, yeah, you need to seek urgent medical advice!" (Oxford Midwife)
- "Midwives sometimes struggle to pass a message to a doctor, this can help...escalation will be improved with the tool particularly for junior midwives. Senior midwives are happier to escalate." (Oxford Midwife)

**Birmingham implementation concerns:**

- "People not escalating a pathological CTG and waiting for 20 minutes... Understanding the purpose and the value of the tool... Training will be important as a lot of midwives don't use regularly the F*it4Labour* tool, so they need to understand it in all areas." (Birmingham Midwife)

**Buckinghamshire evidence-based escalation:**

- "I think it will help with confidence of midwives to escalate something abnormal and know that you've kind of got the risk behind you...I want you to come and look at this." (*Buckinghamshire* Midwife)
- "The *Fit4labour* tool is one language, isn't it?... at work you say things like I'm just not happy with it [speaking about a pathological CTG]. And it's hard to put quite into words why you're not happy with it, but you just don't like it" (*Buckinghamshire* Midwife)

### TECHNOLOGY ADOPTION AND IMPLEMENTATION DOMAIN

#### Theme 5: Training and Digital Confidence

Training emerged as critical across all sites, with Birmingham showing particular emphasis on digital literacy barriers and champion models.

**Oxford baseline concerns:**

- "It needs mandatory training and more reassurance to not miss things on the tool before using it. People always anxious with new technology." (Oxford Midwife)
- "We will need training to use it properly." (Oxford Doctor)

**Birmingham comprehensive training insights:**

- "Think people that struggle with technology will still struggle with this because their confidence with IT is low. It's not that this is difficult to use, it's that their confidence is low." (Birmingham Midwife)
- "Potentially you will need somebody that trains the trainer who is like a champion... have champions in areas within the trust to help those people that are struggling..." (Birmingham Midwife)
- "You also need a field trip out of managers and seniors to a hospital that is using it. To get different clinicians coming in, seeing it being used..." (Birmingham Midwife)
- "It's mandatory for everyone who does obstetrics to attend... once a year." (Birmingham Doctor)

**Training rationale:**

- "People [doctors and midwives] just like to know the rationale, and understand how it works, don't they?" (*Buckinghamshire* Midwife)
- "So as long as we know how to explain this risk information to patient and have time to do so. Training the staff to do so is really important." (*Buckinghamshire* Doctor)

#### Theme 6: Ease of Use and Usability

Validation sites consistently highlighted the tool's intuitive design, confirming successful co-development outcomes.

**Oxford first trial:**

- "Quick to pick up...and then second and third and fourth you're like I know what I'm doing" (Oxford Midwife)
- "Very intuitive and straightforward." (Oxford Midwife)
- "Works for tired brains with colds." (Oxford Midwife)
- "I can read it really clearly, I'm dyslexic." (Oxford Midwife)
- "It is nice. It's easy to use. You know, people will get it very quickly." (Oxford Doctor)

**Birmingham usability validation:**

- "Think considering that we haven't had any training on it, it was relatively easy to use, you know." (Birmingham Midwife)
- "It's easy to use. You don't need much. I mean, the only thing I'd say is obviously you have the established and the non-established labour and it was beautifully written." (Birmingham Doctor)
- "The general demographics were easy to put in and easy to use." (Birmingham Doctor)

**Buckinghamshire** **intuitive design confirmation:**

- "Well, I've had no training on it, but it's very easy to use. Okay. It's very straightforward." (Buckinghamshire Doctor)
- "First impression that it was intuitive." (*Buckinghamshire* Doctor)
- "It felt quite intuitive. Like once you kind of had the information you needed to put down, it felt... especially the first bit with the risk factors, it felt clear where stuff was going." (Buckinghamshire Midwife)

#### Theme 7: Technology Infrastructure and Implementation Barriers

Infrastructure concerns remained consistent across sites, revealing practical deployment challenges.

**Oxford practical concerns:**

- "I think problems to implementation can be internet and chargers always disappear. Computer systems..." (Oxford Midwife)

**Birmingham detailed infrastructure analysis:**

- "It takes a long time, I mean weeks, for the IT team to provide support when devices are not working after reporting... And I think there's two Wi-Fi networks along the labour ward, some of the rooms the Wi-Fi doesn't work, and you can't see the CTGs." (Birmingham Midwife)
- "Also, need to consider charging stations - tablets will go missing and non-charged and then doesn't work. You may need to have restricted access, and one tablet is only used by the manager in charge of the shift." (Birmingham Midwife)
- "Well, we don't have enough computers in the unit let alone plugs to chargers for iPads...We have issues whether or not the CTGs link to the right room or we are using paper copies... compatibility would be the main issue." (Birmingham Doctor)

**Buckinghamshire infrastructure readiness:**

- "We are paperless. We all work on tablets. Yeah. So that won't be a problem." (*Buckinghamshire* Midwife)

#### Theme 8: Digital Overload and Change Management

Concerns about technology proliferation emerged particularly at validation sites, revealing organisational adaptation challenges.

**Birmingham change management insights:**

- "…this is another piece just adding to the workload, which they don't want added to... you've got another task added onto the system and then people have already so many different tabs actually not using them effectively...yeah potentially people are just going to want to bypass it." (Birmingham Midwife)
- "Nobody likes change... you're going to need a lot of buy-in from, a lot of senior people... once those small group of people had it, then everyone went like this, and it'll snowball. You just need to get those first like the iPhone when they came into the market no one liked them." (Birmingham Doctor)

**Buckinghamshire** **organisational readiness:**

- "Culture of [Buckinghamshire Hospital] will be another technology but if it will benefit patients and clinicians will be happy to have it... This is to save baby lives you can see the benefit." (Buckinghamshire Midwife)
- "The staff culture from Buckinghamshire will be accepted but will also see it as another work thing to add to the pile." (*Buckinghamshire* Doctor)

### PATIENT SAFETY AND DECISION-MAKING DOMAIN

#### Theme 9: Decision-Making Safety Net

This theme showed strong and consistent resonance across all sites, with the "co-pilot" concept providing a framework for understanding the tool's supportive role.

**Oxford foundation:**

- "*Fit4Labour* is like a co-pilot in a plane." (Oxford Midwife)
- "Useful in grey areas of CTG interpretations." (Oxford Midwife)
- "Helpful to make difficult decisions... the extra value is the reassurance for the clinician." (Oxford Doctor)
- "That someone very junior has to in their hands so *Fit4labour* is like a safety net." (Oxford Doctor)

**Birmingham clinical decision support:**

- "So, I think it's... it will serve as an aid. It's supposed to help your clinical management. It shouldn't be your final decision. And I like the fact that the physician still has the ultimate decision making." (Birmingham Doctor)
- "So, this will help counselling women who are trying to labour outside of guidelines that want home birth with their three previous sections." (Birmingham Doctor)

**Buckinghamshire uncertainty management:**

- "Like, when you are not too sure in some of these cases where there's a bit of uncertainty, I think this tool will help... that has probably a good role for that." (Buckinghamshire Doctor)
- "Sell it to the users and patients as empowerment tool that helps outcomes and people will like it." (*Buckinghamshire* Doctor)

#### Theme 10: Risk Communication with Patients and Intervention Requests

Patient communication emerged as a significant theme in validation sites, showing sophisticated evolution in clinical thinking.

**Oxford risk communication and intervention concerns:**

- "Patients need to accept their risks even if they don't like to hear it" (Oxford Doctor)
- "We (doctors) are not informing the patients of their real weight of their risks because we don't know, but this is what *Fit4labour* can bring to the table." (Oxford Doctor)
- "Doctors in general are still patronising and make decisions for their patients, this can help minimising it." (Oxford Doctor)
- "In most of the situations is perfectly fine but you know it may change the woman's choices, and she will ask for a c-section delivery. But it will be an objective patient choice. Needs to carefully maintain low false positives to prevent unnecessary c-sections." (Oxford Doctor)
- "I am concern of increasing number of c-sections but for the price of informative and honest communication of risk." (Oxford Doctor)
- "It will be time consuming as you need to spend time with them to explain that numbers... I think patients don't understand risk" (Oxford Doctor)

**Birmingham communication support:**

- "I like is the guidance to how you can inform the patient. Because I think sometimes it's not everyone who wants a figure. It's actually asking... what does this mean? Does it mean that I can labour?" (Birmingham Doctor)
- "Patient are asking for their individual risk more and more in antenatal clinic. And it was actually nice to see this is the background risk of other people and this one is now your risk." (Birmingham Doctor)
- "I think it can increase in caesarean section rates if you're telling someone you've got four times that background risk... also how do we agree with the acceptable risk everyone will have a different threshold." (Birmingham Doctor)

**Buckinghamshire** **comprehensive risk communication:**

- "I think if you've got the information, you should be transparent with them (patients), but you need to be careful how you're wording it to avoid increasing c-sections unnecessarily... it's at this moment in time a baby similar to your baby has this risk but anything can change." (Buckinghamshire Midwife)
- "I don't think patients actually recognise their own risks during pregnancy, so this sort of highlighting's and gives them some actual insight into their pregnancy." (*Buckinghamshire* Doctor)
- "If I was a patient, I would want to know my chances of having a healthy baby vaginally or by a c-section or an instrumental at the onset point of induction." (Buckinghamshire Doctor)
- "May increase c-sections for the price of informed patient choice. Patients think c-section is the safer option often mislead by social media; the *Fit4labour* can play an important role addressing this by supporting informed decisions and evidence-based risk information and potentially reduce unnecessary maternal request c-sections." (*Buckinghamshire* Doctor)

#### Theme 11: Clinical Autonomy and Professional Responsibility

Concerns about maintaining clinical autonomy and medico-legal responsibility were consistently addressed across all sites.

**Oxford autonomy concerns:**

- "I don't want to be told what to do by a machine." (Oxford Midwife)
- "It will support more decisions then make decisions for us." (Oxford Doctor)
- "For me it's an ethical problem of where you draw the line of the risk being too high. You know, because you have all this cases, where does it become red? Where does it become orange? Is ethical to make this difficult call to decide which baby dies and which baby is healthy." (Oxford Doctor)
- "This may help. Maternity needs urgent help in United Kingdom - staff works in a culture of fear and clinicians practice a defensive medicine." (Oxford Doctor)

**Birmingham balanced integration:**

- "It needs to be rolled out very clearly that is not like antenatal score DR [Dawes-Redman system] your clinical judgment is always going to be important... see the patient beyond the technology." (Birmingham Midwife)
- "Maternity currently in the public eye is quite negative because of all the reports of serious incidents and poor care have come out. Also, social media doesn't help. People think doctors want to induce you. Trust and relationship broken. Fit 4labour can decrease this." (Birmingham Midwife)
- "It can deskill people as well, because people will wait to look at the report." (Birmingham Doctor)
- "I worry that it's another tick box exercise, oh, computer says add this number and you're not then needing to use your clinical judgement. And if you've got 20 patients to see and the machine says it's green…you will take shortcuts after a while." (Birmingham Doctor)

**Buckinghamshire** **holistic integration:**

- "Has a huge potential but it is not the solution for everything, and I think as long as people see it as it's a piece of the jigsaw... Because at the end of the day, you are the doctor that knows the person at the end of the bed, not your computer, and it's never ever going to replace that." (*Buckinghamshire* Doctor)

#### Theme 12: System-wide Implementation and Organisational Adoption

Advanced implementation themes emerged only in validation sites, indicating tool maturity and readiness for broader deployment.

**Birmingham initial considerations:**

- "Need to be rolled out very clearly with use of clear protocols." (Birmingham Midwife)
- "It will bridge knowledge gaps at beginning of labour... some staff will feel judged about their clinical practice." (Birmingham Midwife)
- "You will need to have antenatal education for the women prior to labour about it." (Birmingham Midwife)
- "Doctors will need guidance as patients can come back after ending up with a poor outcome say, oh, my risk was 71% and you didn't act on it why?" (Birmingham Doctor)

**Buckinghamshire strategic implementation planning:**

- "I think it would... need to be you either use it in the whole hospital or you don't use it. Again, you haven't got that one language to talk to because someone's going to have you run the fit for labour and someone else hasn't." (*Buckinghamshire* Midwife)
- "So, it’s talking about it and preparing implementation for months in advance." (*Buckinghamshire* Midwife)

# **Supplementary Analysis 2: Site-Specific Thematic Analysis**

### Oxford University Hospitals (Co-development Site)

**Dominant Themes:**

1. **Ease of Use and Usability** - Primary focus on interface design and navigation
2. **Decision-Making Safety Net** - Emergence of "co-pilot" metaphor
3. **Risk Factors and Clinical Comprehensiveness** - Initial identification of limitations
4. **Clinical Autonomy and Professional Responsibility** - Initial resistance to technology

**Key Characteristics:**

- Focus on foundational usability issues
- Emergence of collaborative technology metaphors
- Initial concerns about clinical autonomy
- Recognition of screening potential

### Birmingham Women's and Children's Trust (First Validation Site)

**Dominant Themes:**

1. **Training and Digital Confidence** - Emphasis on digital literacy barriers
2. **Technology Infrastructure and Implementation Barriers** - Detailed practical concerns
3. **Digital Overload and Change Management** - Technology proliferation concerns
4. **Risk Communication with Patients** - Sophisticated discussion of patient counselling

**Key Characteristics:**

- Comprehensive workflow integration planning
- Detailed infrastructure requirements
- Champion training model development
- Patient communication strategy evolution

### Buckinghamshire Hospital (Second Validation Site)

**Dominant Themes:**

1. **System-wide Implementation and Organisational Adoption** - Strategic planning focus
2. **Risk Communication with Patients** - Advanced patient partnership approaches
3. **Clinical Autonomy and Professional Responsibility** - Mature technology integration
4. **Screening and Triage Effectiveness** - Implementation-ready applications

**Key Characteristics:**

- Strategic implementation planning
- Organisational readiness assessment
- Advanced risk communication strategies
- Sophisticated technology-clinical integration

# **Supplementary Analysis 3: Professional Group Stratified Analysis**

### **MIDWIVES' PERSPECTIVES**

**Consistent Priorities Across Sites:**

- Interface design and ease of use
- Triage and screening applications
- Escalation support and communication
- Workflow integration challenges
- Training and support requirements

**Representative Quotes:**

- "It will be helpful for midwives' job as they are the gatekeepers to obstetrics" (Oxford)
- "People that struggle with technology will still struggle with this because their confidence with IT is low" (Birmingham)
- "I think the colours are helpful for people as like a visual aid" (*Buckinghamshire*)

### **DOCTORS' PERSPECTIVES**

**Consistent Priorities Across Sites:**

- Clinical decision-making support
- Risk communication and patient counselling
- Professional autonomy considerations
- Implementation strategy planning
- Medico-legal implications

**Representative Quotes:**

- "It will serve as an aid. It's supposed to help your clinical management. It shouldn't be your final decision" (Birmingham)
- "Has a huge potential but it is not the solution for everything" (Buckinghamshire)
- "This may help. Maternity needs urgent help in United Kingdom" (Oxford)

### **PROFESSIONAL GROUP CONVERGENCE**

**Areas of Agreement:**

- Tool's value as screening and triage aid
- Recognition of "co-pilot" supportive function
- Need for comprehensive training programs
- Infrastructure requirements for implementation
- Importance of maintaining clinical judgment

**Areas of Emphasis Difference:**

- Midwives emphasized:
  - Operational workflow integration
  - User interface and accessibility
- Doctors emphasized:
  - Strategic clinical applications
  - Risk communication and patient counselling

All participant quotes are authentic transcriptions from focus groups and interviews conducted at the respective sites during the study period December 2022 - May 2025.

**Multimedia Appendix 1.** (Video) Demonstration of the *Fit4Labour* tool showing clinical workflow and user interface. Available at: <https://youtu.be/_2VvFMFy_wI>

This video provides a demonstration of the *Fit4Labour* digital decision-support tool, illustrating the clinical workflow from initial patient data entry through risk assessment report generation. The demonstration showcases the user interface design, key features, and clinical applications tested during the usability evaluation study.

**Duration :** 2 minutes 55 seconds

**
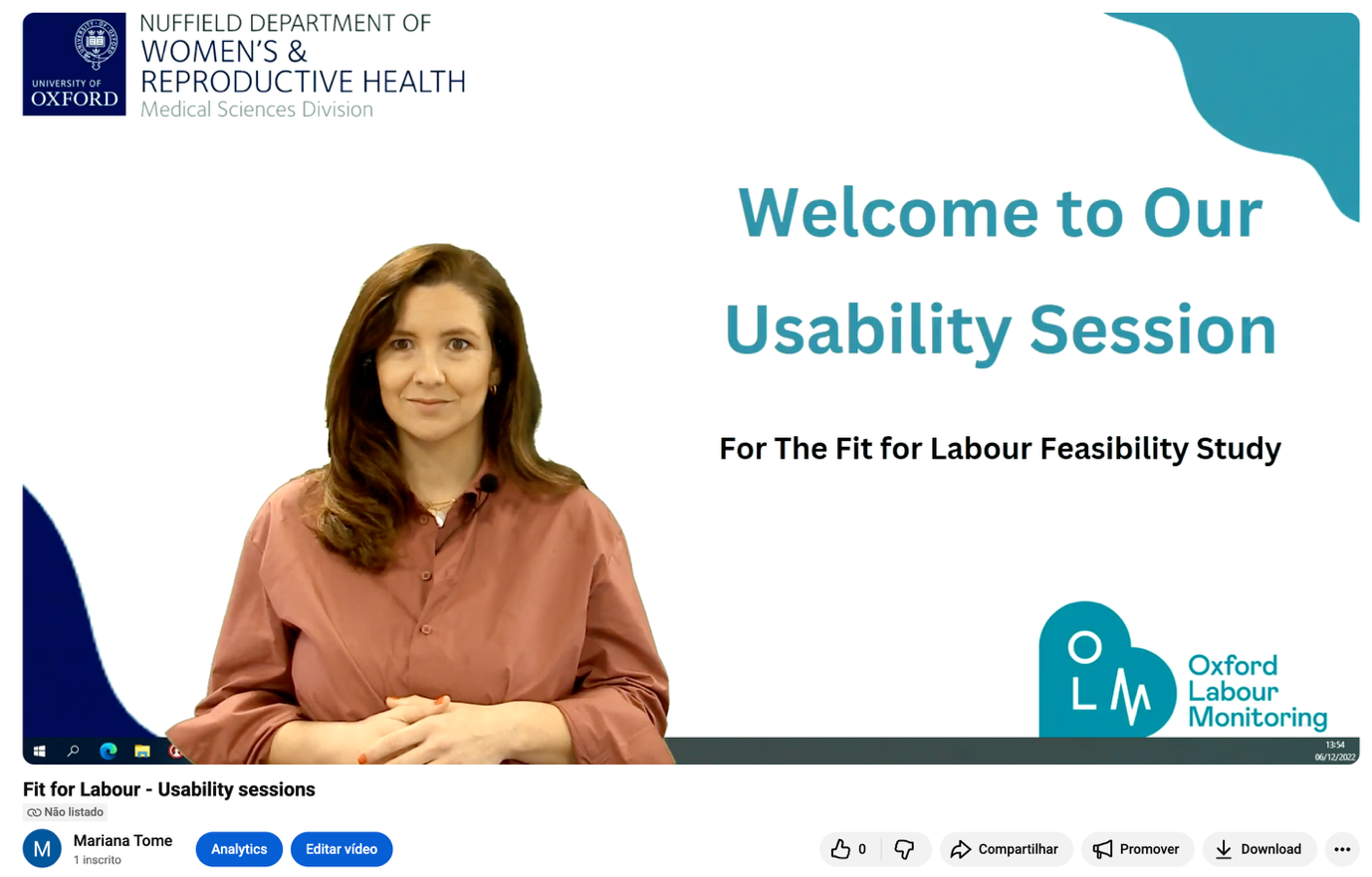
**
